# Steroids-producing nodules: a two-layered adrenocortical nodular structure as a precursor lesion of cortisol-producing adenoma

**DOI:** 10.1016/j.ebiom.2024.105087

**Published:** 2024-04-02

**Authors:** Tazuru Fukumoto, Hironobu Umakoshi, Norifusa Iwahashi, Tatsuki Ogasawara, Maki Yokomoto-Umakoshi, Hiroki Kaneko, Masamichi Fujita, Naohiro Uchida, Hiroshi Nakao, Namiko Kawamura, Yayoi Matsuda, Ryuichi Sakamoto, Takashi Miyazawa, Masahide Seki, Masatoshi Eto, Yoshinao Oda, Yutaka Suzuki, Seishi Ogawa, Yoshihiro Ogawa

**Affiliations:** a Department of Medicine and Bioregulatory Science, Graduate School of Medical Sciences, Kyushu University, Fukuoka, Japan; b Department of Computational Biology and Medical Sciences, Graduate School of Frontier Sciences, The University of Tokyo, Chiba, Japan; c Department of Urology, Graduate School of Medical Sciences, Kyushu University, Fukuoka, Japan; d Department of Anatomic Pathology, Graduate School of Medical Sciences, Kyushu University, Fukuoka, Japan; e Department of Pathology and Tumor Biology, Graduate School of Medicine, Kyoto University, Kyoto, Japan

**Keywords:** Adrenal androgen, Adrenocortical tumorigenesis, Clonal expansion, Cortisol-producing adenoma, Cushing's syndrome, *GNAS*

## Abstract

**Background:**

The human adrenal cortex consists of three functionally and structurally distinct layers; zona glomerulosa, zona fasciculata (zF), and zona reticularis (zR), and produces adrenal steroid hormones in a layer-specific manner; aldosterone, cortisol, and adrenal androgens, respectively. Cortisol-producing adenomas (CPAs) occur mostly as a result of somatic mutations associated with the protein kinase A pathway. However, how CPAs develop after adrenocortical cells acquire genetic mutations, remains poorly understood.

**Methods:**

We conducted integrated approaches combining the detailed histopathologic studies with genetic, RNA-sequencing, and spatially resolved transcriptome (SRT) analyses for the adrenal cortices adjacent to human adrenocortical tumours.

**Findings:**

Histopathological analysis revealed an adrenocortical nodular structure that exhibits the two-layered zF- and zR-like structure. The nodular structures harbour *GNAS* somatic mutations, known as a driver mutation of CPAs, and confer cell proliferative and autonomous steroidogenic capacities, which we termed steroids-producing nodules (SPNs). RNA-sequencing coupled with SRT analysis suggests that the expansion of the zF-like structure contributes to the formation of CPAs, whereas the zR-like structure is characterised by a macrophage-mediated immune response.

**Interpretation:**

We postulate that CPAs arise from a precursor lesion, SPNs, where two distinct cell populations might contribute differently to adrenocortical tumorigenesis. Our data also provide clues to the molecular mechanisms underlying the layered structures of human adrenocortical tissues.

**Funding:**

10.13039/501100001691KAKENHI, 10.13039/100008732The Uehara Memorial Foundation, 10.13039/501100005928Daiwa Securities Health Foundation, 10.13039/501100007206Kaibara Morikazu Medical Science Promotion Foundation, 10.13039/501100004298Secom Science and Technology Foundation, ONO 10.13039/501100009187Medical Research Foundation, and 10.13039/100008695Japan Foundation for Applied Enzymology.

**Research in context**

**Evidence before this study**

The human adrenal cortex consists of three functionally and structurally distinct layers; zona glomerulosa, zona fasciculata (zF), and zona reticularis (zR), and produces adrenal steroid hormones; aldosterone, cortisol, and adrenal androgens, respectively. Although cortisol-producing adenomas (CPAs), a leading cause of adrenocortical tumours, are mostly caused by somatic mutations in genes such as *GNAS* or *PRKACA*, how CPAs arise from a precursor lesion with genetic mutations in adrenocortical tissues remains to be elucidated.

**Added value of this study**

In this study, we report an adrenocortical nodular structure that exhibits a two-layered zF- and zR-like structure. The nodular structures harbour *GNAS* somatic mutations known to confer cell proliferative and autonomous steroidogenic capacities, which we termed “steroids-producing nodules (SPNs)”. Genomic analysis suggests that adrenocortical cells with *GNAS* mutations expand clonally to form SPNs as a result of positive selection in adrenocortical tissues. RNA-sequencing combined with spatially resolved transcriptome analysis suggests that the expansion of zF-like structure contributes to the formation of CPAs, while the zR-like structure might exhibit a macrophage-mediated immune response. In the database analysis of patients with adrenocortical carcinoma, those with higher expression of genes upregulated in the zR-like structure have a better prognosis than with lower expression, suggesting the antitumour potential of the zR-like structure.

**Implications of all the available evidence**

This study suggests that adrenocortical cells, when *GNAS* is mutated, acquire proliferative and autonomous steroidogenic capacities to become dominant as a result of positive selection in adrenocortical tissues, where they expand clonally to form SPNs. SPNs exhibit a two-layered zF- and zR-like structure, where two distinct cell populations might contribute differently to adrenocortical tumorigenesis. Given that *GNAS* mutations found in SPNs are known as a driver mutation of CPAs, we postulate that SPNs are a precursor lesion of CPAs; CPA might arise from one of the SPNs in adrenocortical tissues. Our findings provide clues to understand the underlying mechanisms of the early process of human adrenocortical tumorigenesis and even the molecular mechanisms of the layered structures of human adrenocortical tissues.

## Introduction

The human adrenocortical tissue consists of three functionally and structurally distinct zones, from the outer to the inner layers; zona glomerulosa (zG), zona fasciculata (zF), and zona reticularis (zR), and secretes a range of steroid hormones in a layer-specific manner; aldosterone, cortisol, and adrenal androgens, respectively. The adrenocortical tumours occur mostly as clinically benign adenomas, and occasionally have steroidogenic capacities, such as cortisol-producing adenomas (CPAs) and aldosterone-producing adenomas (APAs). Recent studies have identified somatic gene mutations responsible for the development of adrenocortical tumours.^1^ Indeed, CPAs are a leading cause of adrenocortical tumours, the majority of which are caused by somatic mutations in genes such as *GNAS* or *PRKACA*.^2^^,^^3^ These genes, which, when mutated, result in constitutive activation of the protein kinase A (PKA) pathway to induce cell proliferation and autonomous steroidogenesis,^2^ thereby leading to the development of adrenocorticotropic hormone (ACTH)-independent Cushing's syndrome (CS) and mild autonomic cortisol excess (MACE). However, the mechanism of human adrenocortical tumorigenesis is insufficiently studied; this may be partly because there are no appropriate rodent models of functional adrenocortical tumours. To understand how adrenocortical tumours develop in humans, it is, therefore, essential to analyse human adrenocortical tumour tissues.

Recent breakthroughs in high-throughput DNA sequencing have revealed that somatic gene mutations in a variety of human solid tumours, and interestingly, somatic clonal lineages with individual driver mutations have been found to extend not only to tumour regions but also to pre-neoplastic regions and even apparently normal tissues close to the tumours.^4^ In the adrenocortical tissues, aldosterone-producing micronodules (APMs), also known as aldosterone-producing cell clusters (APCCs), are a nodular lesion of clonal cell populations that harbour somatic mutations associated with APAs and are often found in adrenal cortices adjacent (Adj.AC) to APAs.^5^^,^^6^ We have recently characterised APMs at single-cell resolution and provided evidence that APMs, which develop from zG, occur as a precursor lesion of APAs.^7^ However, there have been no such precursor lesions reported for CPAs. We have speculated that autonomous cortisol-producing cells expand clonally as a precursor lesion of CPAs.

Here, we report an adrenocortical nodular structure, which exhibits a unique two-layered zF- and zR-like structure. The nodular structures harbour *GNAS* somatic mutations known to confer cell proliferative and autonomous steroidogenic capacities in the absence of ACTH stimulation, which we have termed “steroids-producing nodules (SPNs)”. RNA-sequencing (RNA-seq) combined with spatially resolved transcriptome (SRT) analysis reveals that the expansion of zF-like structure contributes to the formation of CPAs, whereas the zR-like structure might exhibit a macrophage-mediated immune response. This study suggests that CPAs arise from a precursor lesion, SPNs, where two distinct cell populations might differently contribute to adrenocortical tumorigenesis. Our data also help elucidate the molecular mechanisms underlying the layered structures of human adrenocortical tissues.

## Methods

### Ethics

This study was approved by the Institutional Review Board of Kyushu University (approval study number: 21025-01) and was conducted according to the guidelines for clinical studies published by the Ministry of Health and Labor, Japan. Written informed consent was obtained from all participants.

### Study design and participants

This study was conducted at Kyushu University Hospital, a referral centre in Japan. We identified patients with adrenal CS, primary aldosteronism (PA), and nonfunctioning adrenal tumours (nonfunctioning adrenocortical adenoma [NFA], adrenal ganglioneuroma [GN], and adrenal myelolipoma [ML]) who underwent adrenalectomy at Kyushu University Hospital between 2014 and 2020. Diagnosis of the diseases was confirmed based on the Endocrine Society clinical practice guidelines.^8^^,^^9^ In patients with adrenal CS, PA, and NFA, immunostaining for 11β-hydroxylase (CYP11B1), aldosterone synthase (CYP11B2), and 17α-hydroxylase/17,20-lyase (CYP17A1) confirmed the presence of CPAs, APAs, and NFAs. In 74 resected samples, the paired adrenal tumours-adjacent adrenocortical tissue samples were available (CPA, n = 12; APA, n = 56; NFA, n = 2; GN, n = 2; ML, n = 2). Sex was recorded based on self-report by patients and patients were included irrespective of their sex.

### Statistics

Statistical analyses of clinical characteristics were performed with EZR (Saitama Medical Center, Jichi Medical University, Saitama, Japan), a graphical user interface for R (The R Foundation for Statistical Computing, Vienna, Austria, version 3.5.2) that is a modified version of R commander (version 2.5–1) designed to add statistical functions frequently used in biostatistics.^10^ Continuous variables were expressed as the mean ± standard deviation for normally distributed data and as the median and interquartile range for non-normally distributed data.

### Sample size estimation

No power analysis or sample size calculation were conducted prior to the onset of the study. Samples were determined by cases undergoing adrenalectomy during the specified period and for whom specimens were available.

### Tissue preparation

Serial sections (4-μm) from formalin-fixed paraffin-embedded (FFPE) specimens were analysed with Haematoxylin and eosin (HE) and immunohistochemistry. We examined key steroidogenic enzymes, CYP11B1, CYB11B2, and CYP17A1, in 74 FFPE specimens whose adjacent adrenocortical tissue samples were available. In some samples, we performed staining for 3β-hydroxysteroid dehydrogenase (HSD3B2), cytochrome b5 type A (CYB5A), 21-hydroxylase (CYP21A2), and cytochrome P450 11A1 (CYP11A1).

### Immunohistochemistry and immunofluorescence

Immunohistochemistry was performed using antibodies against CYP11B1 (rat monoclonal; 1:300, Millipore, #MABS502, RRID:AB_2920814), CYP17A1 (rabbit monoclonal; 1:250, Abcam, #ab134910, RRID:AB_2895598), CYP11A1 (rabbit polyclonal; 1:150, Atlas Antibodies, #HPA016436, RRID:AB_1847423), CYB5A (mouse monoclonal; 1:1000, OriGene, #AM31963PU-N, RRID:AB_2940799), CYP21A2 (rabbit polyclonal; 1:1000, Atlas Antibodies, #HPA048979, RRID:AB_2680584), NR5A1 (mouse monoclonal; 1:600, Perseus Proteomics, #PP-N1655-0C, RRID:AB_2904221), and HSD3B2 (provided by Dr. C.E. Gomez-Sanchez [University of Mississippi Medical Center, Jackson, MS; RRID: AB_2728753]). For immunofluorescence, the same antibodies and dilutions were used, and images were captured using a Keyence Biozero BZ-X710 microscope.

### DNA and RNA isolation

To isolate nucleic acids, CPAs, APAs, and periadrenal adipose tissue, as a control for germline DNA, were manually cut from the unstained FFPE sections. SPNs and adjacent adrenocortical samples as controls were microdissected from the unstained FFPE sections using a laser microdissection (LMD) microscope (Model LMD6500, Leica Microsystems) guided by the CYP17A1 immunohistochemistry slide. Genomic DNA and RNA were co-extracted from SPNs, CPAs, APAs, and adrenocortical tissue samples using the AllPrep DNA/RNA FFPE kit (Qiagen, 80234) according to the manufacturer's instructions. DNA was used for targeted capture and whole-exome sequencing, while RNA was used for RNA-seq analysis. The RNA fragment size was analysed using an RNA 6000 Pico Kit (Agilent Technologies, 5067-1513) running on a 2100 Bioanalyzer (Agilent Technologies). DV200 values, representing the percentage of RNA fragments >200 nucleotides in length, were determined according to the manufacturer's instructions. The RNA quality was assessed using DV200 values, and the sample with the lowest DV200 was 21%.

### Targeted capture sequencing and somatic mutation detection

Targeted capture sequencing was performed using xGen Predesigned Gene Capture Pools (IDT), followed by sequencing of enriched fragments on a DNBSEQ-G400RS (MGI) in 100-bp paired-end mode with an average depth of 723 × (42–2703×). We selected 36 genes from the custom bait library, including CS/MACE-associated genes. Sequence alignment and mutation calling were performed using the Genomon2 pipelines (https://genomon.readthedocs.io/ja/latest/). To call somatic mutations, we used the filtering parameters for variant calling as follows: (i) A mapping quality score ≥20, (ii) a base quality score ≥15, (iii) a number of variant reads in the tumour ≥8, (iv) variant allele frequency (VAF) in the tumour ≥0.05, (v) an empirical Bayesian mutation calling (EBCall) P value ≤ 1.0 × 10^−4^, and (vi) variants presenting in bidirectional reads. Conversely, we excluded the following variants: Synonymous single nucleotide variants (SNVs), known variants listed in the 1000 Genomes Project (May 2011 release), Exome Sequencing Project (ESP) 6500, and the Human Genome Variation Database (HGVD; October 2013 release) with frequencies >0.001. Finally, mapping errors were removed by visual inspection using Integrative Genomics Viewer.

### Whole-exome sequencing (WES)

WES was performed on two SPNs (N5, N6) and one CPA, and adipose tissue was used as a germline DNA control in a single case (Case C-5). WES libraries were prepared using the xGen Exome Research Panel v2 (IDT), followed by sequencing of enriched exon fragments on a DNBSEQ-G400RS (MGI) in 100-bp paired-end mode with an average depth of 19 × (176–200×) for SPNs, CPA, and germline DNA. Sequence alignment and mutation calling were performed using Genomon2 pipelines (https://genomon.readthedocs.io/ja/latest/) as previously reported.^11^ Briefly, sequencing reads were aligned to the human genome reference (GRCh37) using the Burrows-Wheeler Aligner (version 0.7.8) with default parameter settings. PCR duplicates were eliminated using Biobambam (version 0.0.191) (https://github.com/gt1/biobambam). Somatic mutations were detected by eliminating polymorphisms and sequencing errors. To achieve this, Genomon2 first discards low-quality, unreliable reads and variants according to the following criteria: (i) Mapping quality <20 and (ii) base call quality <15; variants were further filtered by the following criteria: (iii) both tumour and normal depths ≥8, (iv) number of variant reads in tumour ≥4, (v) number of variant reads in normal ≤1, (vi) VAFs in tumour ≥0.05, (vii) VAFs in germline control ≤0.02 (viii) Fisher's exact test P value < 0.1, and (ix) presenting in bidirectional reads. To select variants that were observed at significantly higher VAFs than expected for errors, we used the following criteria: (x) P value ≤ 1.0 × 10^−4^, for which significance is evaluated by the EBcall algorithm^12^ based on an empirical distribution of VAFs as determined using the WES data of non-paired germline samples (n = 18). Candidate mutations were visually inspected using Integrative Genomics Viewer to further eliminate sequencing errors.

### RNA-seq analysis of laser capture microdissection (LCM)- and manually dissected FFPE specimens

RNA-seq analysis was performed using total RNA (50 ng) obtained from SPNs (n = 5), CPAs (n = 6), adrenocortical samples adjacent to CPA (n = 7), and adrenocortical samples adjacent to non-CPA (n = 6). Libraries were prepared using the SMART-Seq Stranded Kit (Takara Bio, 634444), quantified using a Bioanalyzer DNA-sensitivity kit (Agilent Technologies, 5067-4626), and sequenced on the NextSeq 500 (Illumina) using a 36-cycle paired-end protocol, providing approximately 33 million reads per sample. Base call files were converted to the FASTQ format using Bcl2Fastq (Illumina). All reads were aligned to the reference genome (human, hg19) using the 89 CLC genomics workbench (version 10.1.1), and gene expression was quantified.

### RNA-seq data analysis

Gene expression data were used as inputs for the R package edgeR (version 3.40.2).^13^ Genes with low expression levels were excluded using the filterByExpr function. Gene counts were normalised by applying the trimmed mean of the M-values normalisation method using the calcNormFactors and cpm functions. The resulting log_2_ counts per million (log_2_CPM) were used as inputs for downstream analyses. Differentially expressed genes (DEGs) were detected using the glmFit and glmLRT functions. The Benjamini-Hochberg method was used to correct for multiple comparisons. DEGs were defined as genes with an absolute value of log_2_ fold-change greater than 0.5 and an adjusted P value of less than 0.1. Gene set enrichment analysis (GSEA) was performed using the R package fgsea (version 1.24.0), and zF and zR gene sets curated from the results of two studies^6^^,^^14^ were examined. Enrichment analysis was performed using Metascape with default parameters. Principal component (PC) analysis was performed using the prcomp function of the R package stats (version 4.2.1), with log_2_CPM data of the top 1000 most variable genes as input.

### Spatial transcriptomic analysis

The FFPE section of Case C-5 was processed using the 10× Genomics Visium Spatial Gene Expression Slide Kit (10× Genomics, PN-1000188), FFPE Reagent Kit (10× Genomics, PN-1000361), and Human Transcriptome Probe Kit (10× Genomics, PN-1000364). Tissue RNA was extracted using the AllPrep DNA/RNA FFPE kit (Qiagen, 80234) and confirmed to have a DV200 of >50%. The tissue section of 6.5 mm square and 5 μm-thick tissue sections were cut and placed on the capture area of the gene expression slide. Libraries were prepared according to the manufacturer's instructions. Sequencing was performed using a DNBSEQ-G400 (MGI). Mapping and counting were performed using Space Ranger (version 1.3.1) with the reference genome GRCh38-2020-A.

### SRT data analysis

#### Normalisation and dimensional reduction

SRT data were processed using the R package Seurat (version 4.3.0).^15^ The unique molecular identifier (UMI) count data were normalised using the regularised negative binomial regression method implemented in the SCTransform function. PC analysis using the RunPCA function was performed with the top 3000 highly variable genes. Uniform Manifold Approximation and Projection (UMAP) was performed using the RunUMAP function with the top 30 PCs.^16^

#### Unsupervised clustering

Unsupervised clustering was performed using the shared nearest-neighbour modularity optimisation-based clustering algorithm with the FindNeighbors and FindClusters functions. The top 30 PCs were used as input for the FindNeighbors function. The optimal value of the resolution parameter of the FindClusters function was selected between 0.4 and 2.0. For the resolution setting, a stability score based on single-cell consensus clustering was used as a reference.^17^ The stability score was calculated using the Clustree function in the R Clustree package.^18^ The resolution parameter was set to 0.9 for clustering of 3380 SRT spots after filtering and 1.4 for the SPN subclustering.

#### Correlation analysis

The average expression levels of the expression-based clusters (adrenal cortex, SPN, and CPA) were calculated using the AverageExpression function of Seurat. Spearman correlation coefficients between clusters were calculated using the rcorr function of R package Hmisc, applied to the scaled data of the top 3000 highly variable genes. Clusters are ordered by hierarchical clustering of the Spearman correlation coefficients.

#### Differential expression analysis

Differential expression analysis was performed using the R package presto wilcoxauc function with normalised gene expression data. The P values were adjusted for multiple testing using the Benjamini-Hochberg's correction. DEGs were defined as those with a log fold change >0.25 and an adjusted P value < 0.05.

#### Deconvolution analysis

Deconvolution analysis of the SRT data was performed using the create.RCTD and run.RCTD functions in the R package Spacexr (version 2.0.6).^19^ The doublet_mode parameter of the run.RCTD function is set to full. Adrenocortical, macrophage, and T-cell data were selected from our previously reported single-cell RNA-seq data of the normal adrenal cortex and used as references.^7^

#### Enrichment analysis

Enrichment analysis of DEGs was performed using Metascape, a web-based portal designed to provide a comprehensive resource for the annotation and analysis of gene lists.^20^ Metascape analysis was conducted using default parameters, while the Gene Ontology Biological Process (GOBP) and Kyoto Encyclopaedia of Genes and Genomes (KEGG) pathways were selected from the significantly overrepresented gene sets.

Single-sample GSEA (ssGSEA) was performed using the enrichment function of R package escape, and enrichment scores were calculated for each gene set per spot.^21^ Hallmark gene sets from the Molecular Signatures Database (MSigDB) and Senescence-associated Mayo (SenMayo), a gene set of the senescence-associated pathway, were used.^22^^,^^23^ For correlation analysis between the normalised enrichment score and macrophage proportion estimated from the deconvolution analysis, a Pearson coefficient >0.3, P value < 0.05 was defined as statistically significant.

#### Pathway activity inference

The R package decoupleR (version 2.2.2) and PROGENy were used to estimate the pathway activity of the SPN subclusters.^24^^,^^25^ PROGENy is a public resource for 14 different pathways, with genes ranked according to their importance in each pathway.^25^ The 100 most important genes in each pathway were selected, and the pathway activity of each spot was calculated using the runwmean function.

#### Trajectory analysis

The developmental trajectory was simulated with dimensionality reduction to a diffusion map using R package destiny (version 3.1.0).^26^ The top 30 PCs were used as inputs, and the diffusion component was calculated using the DiffusionMap function. The main lines of the trajectory were constructed by fitting the computeElasticPrincipalTree function in the R package ElPiGraph.R (version 1.0.0). The pseudotime was calculated from the constructed trajectory. The starting point of the trajectory was set to the location in the adrenal cortex with the smallest DC1 (located at the left end of the diffusion map).

The R package tradeSeq (version 1.14.0)^27^ was used to detect DEGs along the pseudotime. A generalised additive model (GAM) was fitted for each gene with count data for the top 3000 highly variable genes using the fitGAM function with the nknots parameter set to 6. DEGs along the pseudotime in each CPA lineage and SPN lineage were detected using the associationTest function with the l2fc parameter set to 1 and lineage parameter set to TRUE. Genes with adjusted P value <0.05 were considered significant. The detected DEGs along the pseudotime were clustered using the k-means function in the R package stats with the expression data smoothed by GAM as input. The centres parameter was set to 5 for DEGs in the CPA lineage and 4 for the SPN lineage.

### Multiplex immunofluorescence

Multiplex immunofluorescence staining was performed using the PhenoCycler-Fusion System from Akoya Biosciences following the manufacturer's instructions.^28^ The following Akoya antibody barcode reporters were used for staining: CD4-BX003-Cy5 (Akoya Biosciences, 4550112), CD68-BX015-Cy5 (Akoya Biosciences, 4550113), and CD8-BX026-Atto 550 (Akoya Biosciences, 4250012). Each antibody was tagged with a unique oligonucleotide (barcode) and visualised via hybridisation with a fluorescent dye (reporter), to which an oligonucleotide complementary to the barcode was added. Hybridisation and imaging were performed using the PhenoCycler-Fusion platform (Akoya Biosciences). After the PhenoCycler-Fusion run, the output QPTIFF images were imported into QuPath for observation.^29^

### The cancer genome atlas (TCGA) data analysis

RNA-seq data and sample information (overall survival, age, sex, etc.) of 79 adrenocortical carcinoma (ACC) cases from TCGA consortium were obtained using the R package recount3 (version 1.6.0).^30^ For the acquired gene expression data, edgeR was used to exclude low-expressed genes, normalise and identify DEGs as described in the “RNA-seq data analysis” section. Additionally, the transcripts per million (TPM) values were calculated. Survival analysis was performed using the R package survival (version 3.5–3) (https://doi.org/10.1002/sim.956). The survdiff function was used for the log-rank test of Kaplan–Meier survival curve comparison, and the coxph function was used for Cox regression analysis. The proportional hazards assumption underlying Cox regression was assessed using log–log survival curves and scaled Schoenfeld residuals plots. It was considered to be valid when the log–log plot showed that the two curves were parallel and the Schoenfeld residual test did not reject the null hypothesis (P value cutoff 0.05). Kaplan–Meier curves, log–log survival curves, scaled Schoenfeld residuals plots and forest plot of hazard ratios were plotted using the R package survminer (version 0.4.9). GSEA was performed using the R package fgsea (version 1.24.0), and the KEGG pathway gene sets were examined. Deconvolution analysis was performed of the TIMER 2.0.^31^ TIMER 2.0 was run with TPM values as input, and the results of the quanTIseq algorithm were selected from the output of TIMER 2.0.^32^ The respective subpopulations of macrophages, T cells, B cells, NK cells, mast cells, and myeloid dendritic cells were summarised. The Wilcoxon rank sum test was used to compare the proportions of immune cells, and an adjusted P value < 0.05 and a log_2_ fold change in proportion >0.5 were considered statistically significant.

### Role of funders

The funder of this study had no role in data collection, analysis, or interpretation, trial design, patient recruitment, or any aspect pertinent to the study.

## Results

### Identification of a two-layered adrenocortical nodular structure; SPNs

**Fig. 1:**
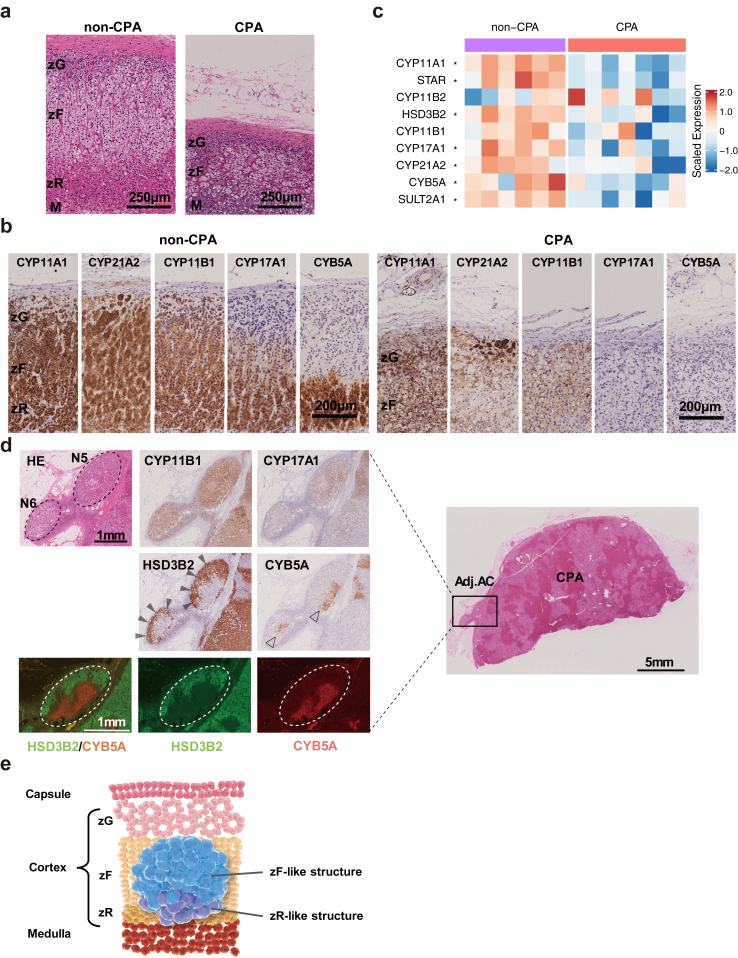
**Identification of SPNs. (a)** Histological analysis of Adj. AC to non-CPA (NFA; left, female and 35 years old) and CPA (right, female and 36 years old; HE staining). Scale bar, 250 μm. (**b**) Representative histological images of IHC staining for the 5 steroidogenic enzymes, CYP11A1, CYP21A2, CYP11B1, CYP17A1 and CYB5A, in Adj.AC to non-CPA (GN; left, male and 25 years old) and CPA (right, male and 44 years old). Scale bar, 200 μm. (**c**) Heatmap comparing expression of key steroidogenic enzyme genes between Adj.AC to non-CPA and Adj.AC to CPA; those with significant expression differences between the two groups are indicated by asterisks. The adjusted P values of the Wilcoxon rank sum test, corrected by the Benjamini-Hochberg method, are as follows: *CYP11A1*: P < 0.0001, *STAR*: P < 0.0001, *HSD3B2*: P value = 0.0016, *CYP17A1*: P value = 0.0061, *CYP21A2*: P value = 0.048, *CYB5A*: P value = 0.019, *SULT2A1*: P value = 0.0026. (**d**) Histological and immunohistochemical evaluation of SPNs in Case C-5. HE and IHC staining of CYP11B1, CYP17A1, HSD3B2 and CYB5A. Staining of HSD3B2 and CYB5A shows that the SPNs form a two-layered structure; an outer zF-like structure (black arrowheads) and an inner zR-like structure (open arrowheads). Scale bar, 5 or 1 mm. (**e**) Schematic representation of SPNs.

To detect lesions that produce steroids autonomously in adrenocortical tissues, we first examined Adj.AC to CPA. We performed histopathological analysis of Adj.AC to CPA (n = 12), using those to non-CPAs (n = 7) as a control. The clinical characteristics of patients with CPA are listed in Table S1. 1-mg dexamethasone suppression test (DST) did not suppress serum cortisol levels et al., and ACTH levels were suppressed. HE staining revealed histological alterations in the layered structure of Adj.AC to CPA, characterised by marked atrophy of zF and loss of most zR (Fig. 1a). Immunohistochemical (IHC) analysis revealed a decline in the expression of steroidogenic enzymes such as CYP17A1, which is essential for cortisol synthesis, and CYB5A, which is required for adrenal androgen synthesis^33^ (Fig. 1b). RNA-seq analysis confirmed the reduced expression of steroidogenic genes in Adj.AC to CPA (Fig. 1c, Fig. S1a, Table S2). These observations suggest that Adj.AC to CPA is characterised by impaired steroidogenesis, with decreased zF and loss of most zR.

Next, we examined Adj.AC to CPA by IHC analysis for the steroidogenic enzymes, CYP11B1 and CYP17A1. In 6 out of 12 adrenal samples, we identified adrenocortical nodular structures, which were stained strongly with anti-CYP11B1 and anti-CYP17A1 antibodies; the identified structures were located beneath zG and close to the lower edge of the cortex (width: 979–2560 μm, depth: 619–2450 μm, area: 0.54–3.73 mm^2^; Fig. 1d, Fig. S1b). IHC analysis of HSD3B2 and CYB5A, which are specific for both zG and zF, and zR, respectively,^33^ revealed that the nodules exhibited a two-layered structure; an outer zF-like structure (HSD3B2-positive, CYB5A-negative cells) and an inner zR-like structure (HSD3B2-negative, CYB5A-positive cells; Fig. 1d, Fig. S1b). Since SPNs are very small in size and most tissue samples are used for nucleic acid extraction, there were sectioned specimens available for immunostaining for both HSD3B2 and CYB5A only in Cases C-2 and C-5. These observations suggest that the zF- and zR-like structures have distinct capacities for steroidogenesis; cortisol and androgens, respectively. We, hereafter, termed the nodular structures “steroids-producing nodules (SPNs)” (Fig. 1e).

### SPNs harbour *GNAS* somatic mutations

**Fig. 2:**
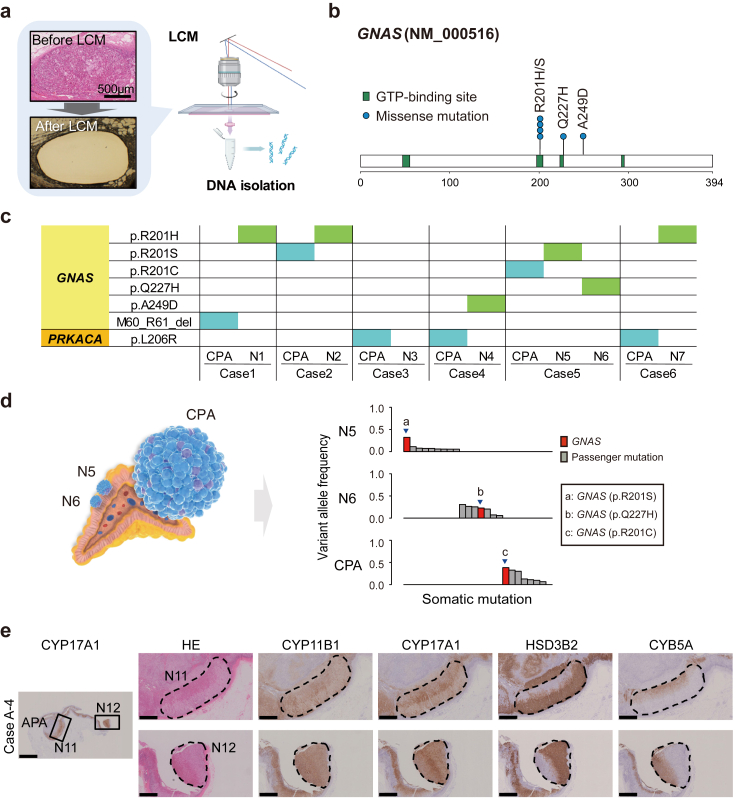
**Genetic analysis of SPNs. (a)** LCM of SPNs in Case C-5 for DNA isolation. Photomicrographs before (upper, HE staining) and after (lower, unstained) LCM confirming isolation of the desired lesion. Scale bar, 500 μm. (**b**) Sites of *GNAS* mutations in SPNs. (**c**) Genetic relationships between SPNs and their corresponding CPAs. (**d**) Somatic mutations in the SPNs (N5, N6) and CPA in Case C-5 as evaluated by WES. (**e**) Histological and immunohistochemical evaluation of SPNs (N11, N12) in Case A-4. HE and IHC staining of CYP11B1, CYP17A1, HSD3B2 and CYB5A. Staining of HSD3B2 and CYB5A shows that the SPNs form a two-layered structure; an outer zF-like structure (black arrowheads) and an inner zR-like structure (open arrowheads). Scale bar, 5 or 1 mm.

We constructed a panel of 36 genes that encompasses mutations known to be associated with adrenocortical tumorigenesis (Table S3) and performed targeted capture sequencing of SPNs. Among the six adrenal samples in which SPNs are identified, seven SPNs (N1–N7; two SPNs in a single adrenal sample) were excised from FFPE sections using LCM to purify genomic DNA (Fig. 2a). In this study, we found that six out of seven SPNs harbour *GNAS* mutations, five of which are R201H, R201S, or Q227H, which have been reported as hotspot mutations in CS (Fig. 2b, Table 1).^2^ Although no mutations were found in N3, the lesion was small (0.55 mm^2^ in Fig. S1b). In the atrophied adrenocortical tissues around the SPNs, no mutations of these 36 genes examined were found. These observations suggest that the *GNAS* somatic mutations confer the autonomous steroidogenic capacity to SPNs. Conversely, no somatic mutations in *PRKACA*, another major driver mutation of CPAs, were identified in SPNs. We also performed targeted capture sequencing to elucidate the genetic relationship between the paired SPNs and CPA in each adrenal sample. Of the six adrenal samples examined, three CPAs harboured somatic mutations in *PRKACA,* and the rest showed *GNAS* somatic mutations. Interestingly, in each adrenal sample, the sites of *GNAS* mutations detected in the CPAs differed from those detected in the paired SPNs (Fig. 2c, Table 1). In addition, WES analysis of Case C-5 with two SPNs (N5, N6) revealed no shared mutations between the paired SPNs and CPA (Fig. 2d), suggesting that the two SPNs and CPA in Case C-5 are of clonally independent origin. Of the six CPAs without SPNs, five CPAs harboured mutations in *PRKACA* and one in *GNAS*.

**Table 1:** Somatic mutations identified in SPNs and corresponding CPAs.

Case	Sample	Gene	Reference allele	Variant allele	Amino acid change	Number of variant reads	Read depth	Variant allele frequency	Variant allele frequency in matched adjacent normal tissue	Reference sequence
C-1	CPA	*GNAS*	GATGAG	–	M60_R61_del	123	545	0.23	0	NM_000516
	N1	*GNAS*	G	A	p.R201H	179	546	0.33	0	NM_000516
C-2	CPA	*GNAS*	C	A	p.R201S	39	150	0.26	0	NM_000516
	N2	*GNAS*	G	A	p.R201H	55	256	0.21	0	NM_000516
C-3	CPA	*PRKACA*	A	C	p.L206R	270	899	0.30	0	NM_002730
	N3	–	–	–	–	–	–	–	–	–
C-4	CPA	*PRKACA*	A	C	p.L206R	308	1270	0.24	0	NM_002730
	N4	*GNAS*	C	A	p.A249D	176	514	0.34	0	NM_000516
C-5	CPA	*GNAS*	C	T	p.R201C	782	2545	0.31	0	NM_000516
	N5	*GNAS*	C	A	p.R201S	663	2065	0.32	0	NM_000516
	N6	*GNAS*	G	T	p.Q227H	662	2703	0.25	0	NM_000516
C-6	CPA	*PRKACA*	A	C	p.L206R	165	436	0.38	0	NM_002730
	N7	*GNAS*	G	A	p.R201H	91	528	0.17	0	NM_000516

Next, we examined whether SPNs occurred in Adj.AC to non-CPAs. Histopathological analysis with CYP11B1, CYP17A1, HSD3B2 and CYB5A revealed that Adj.AC to non-CPA are not atrophic as described above (Fig. 1b). In 4 out of 62 adrenal samples examined, we found five two-layered nodular structures similar to SPNs (N8–N12; two in a single adrenal sample; Fig. 2e, Fig. S2). Targeted capture sequencing revealed that all of five structures harboured *GNAS* somatic mutations (Table 2), suggesting that SPNs occur in Adj.AC to non-CPA as well as those to CPA. The frequency of SPNs in cases with CPA (50.0%) was higher than in those with non-CPA (6.5%; Fisher's exact test, P value = 0.00076, odds ratio 0.069, 95% confidence interval [0.015, 0.35]). Out of four cases with SPNs, only one case (Case A-2) had a clinical diagnosis of PA with MACE. In the other cases, cortisol levels were suppressed by 1-mg DST.

**Table 2:** Somatic mutations identified in the paired SPNs and adrenocortical tumours.

Case	Sample	Gene	Reference allele	Variant allele	Amino acid change	Number of variant reads	Read depth	Variant allele frequency	Variant allele frequency in matched adjacent normal tissue	Reference sequence
A-1	APA	*KCNJ5*	T	G	p.L168R	36	138	0.26	0	NM_000890
	N8	*GNAS*	C	T	p.R201C	101	434	0.23	0	NM_000516
A-2	APA	*CTNNB1*	T	C	p.S45P	34	145	0.26	0	NM_001098209
	N9	*GNAS*	G	A	p.R201H	4	42	0.08	0	NM_000516
A-3	APA	*KCNJ5*	T	G	p.L168R	21	126	0.17	0	NM_000890
	N10	*GNAS*	G	A	p.R201H	170	455	0.37	0	NM_000516
A-4	APA	*KCNJ5*	T	G	p.L168R	109	317	0.34	0	NM_002730
	N11	*GNAS*	G	A	p.R201H	204	619	0.33	0	NM_000516
	N12	*GNAS*	C	T	p.R201C	113	457	0.27	0	NM_000516

### SPNs show gene expression profiles different from those of CPAs

**Fig. 3:**
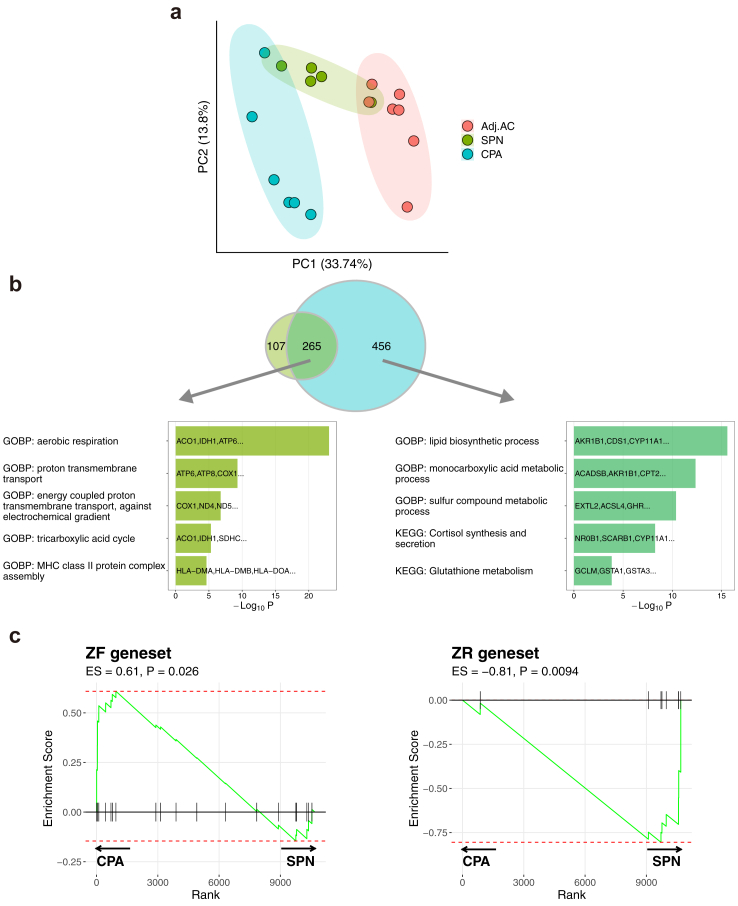
**Transcriptomic analysis of SPNs. (a)** Dot plot showing the result of principal component analysis. Principal component analysis was performed based on the expression data of the top 1000 most variable genes. Dots represent each sample and colours represent tissue type. (**b**) Diagram showing the overlap of genes upregulated in CPAs and SPNs compared with Adj.AC. Gene ontology analysis of the top 10 associated with upregulated genes in both CPAs and SPNs. (**c**) Gene set enrichment analysis of zF and zR gene sets in SPNs and CPAs.

We conducted RNA-seq analysis of SPNs (n = 5), CPAs (n = 6), and Adj.AC to CPA (n = 7). Of the six CPAs, four harboured *GNAS* somatic mutations and two harboured *PRKACA* somatic mutations. In the principal component analysis, SPNs showed gene expression profiles different from those of CPAs and Adj.AC to CPA (Fig. 3a). Both CPAs and SPNs shared 265 genes that are upregulated relative to Adj.AC to CPA as a control, including those involved in steroidogenesis, which is consistent with the IHC analysis data (Figs. 1d and 3b, Fig. S1b, Table S4). In enrichment analysis, the genes commonly upregulated were enriched in the gene sets involved in steroid metabolism, such as “lipid biosynthetic process” and “cortisol synthesis and secretion” (Table S5). The TRRUST database analysis indicated that the commonly upregulated genes were controlled by *NR5A1*, which is critical for adrenal development and differentiated adrenocortical function (Fig. S3a).^34^ There was no significant difference in *NR5A1* expression between CPAs and SPNs (Fig. S3b), consistent with the IHC staining of the *NR5A1*-encoded protein steroidogenic factor 1/adrenal 4 binding protein (SF1/Ad4BP; Fig. S3c).

In this study, 107 genes were upregulated only in SPNs, which are enriched in the gene sets associated with immune response, such as “MHC class II protein complex assembly” and “antigen processing and presentation of peptide antigen” (Fig. 3b, Table S6). The MHC class II protein complex-related gene set is documented to be upregulated in zR.^7^^,^^33^ In contrast, 456 genes were upregulated only in CPAs and were enriched in gene sets associated with cholesterol metabolism and angiogenesis (Fig. 3b, Table S7). In the IHC analysis, SPNs seemed to have a higher proportion of zR-like components and lower proportion of zF-like components than CPAs (Fig. S3d). We also performed GSEA between SPNs and CPAs using the zF- and zR-related gene sets curated from the literature (Table S8).^7^^,^^33^ The enrichment score of the zR-related gene set was higher in SPNs than in CPAs, while that of the zF-related gene set was higher in CPAs (Fig. 3c), consistent with the IHC analysis (Fig. S3d).

### SPNs have high expression of genes associated with adrenal androgen synthesis and immune response

**Fig. 4:**
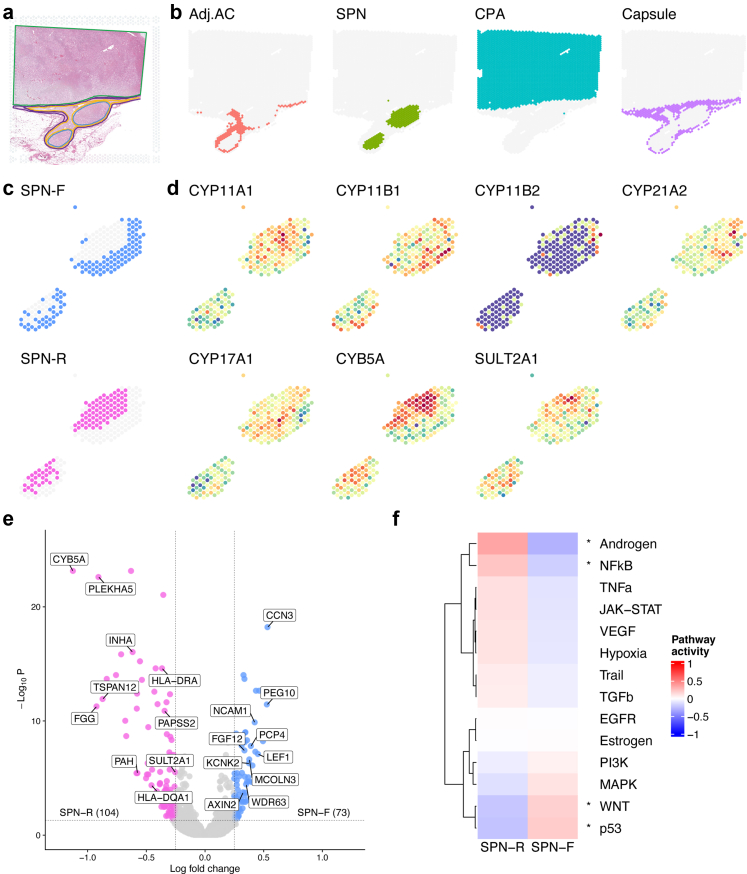
**Spatial transcriptomic analysis of SPNs. (a)** Histological regions annotated by microscopic findings of HE staining. Adj.AC (orange), CPA (green), SPNs (blue) and capsule (purple). (**b**) Split view of the spatial distribution showing the clusters annotated with a combination of unsupervised clustering based on gene expression and histological annotation. (**c**) Split view of the spatial distribution showing the SPN subclusters annotated with a combination of unsupervised clustering based on gene expression and histological annotation. (**d**) Spatial distribution showing gene expression of steroidogenic enzymes. (**e**) Volcano plot showing differential gene expression between SPN-F and SPN-R, with select statically and biologically significant genes being highlighted. Horizontal and vertical dashed lines indicate the adjusted P value threshold and log fold-change threshold, respectively. Genes with log fold-change >0.25 and Benjamini-Hochberg adjusted P value < 0.05 (Wilcoxon rank sum test) were considered significant. (**f**) Heatmap showing the results of pathway activity estimation by the PROGENy method in the SPN-F and SPN-R clusters. Pathways with adjusted P value < 0.05 in the Wilcoxon rank sum test (Benjamini-Hochberg's correction) are marked with an asterisk (Androgen: P value < 0.0001, NFκB: P value = 0.014, WNT: P value = 0.013, p53: P value = 0.013).

Given that SPNs possess a two-layered zF- and zR-like structure, we performed a SRT analysis. In this study, the Visium FFPE pipeline was used to obtain SRT data for the SPNs (N5 and N6) in Case C-5 (Fig. 4a). Transcriptome data were also obtained from the adrenocortical tissues around the SPNs and CPA. SRT data were obtained from 3485 of 5000 spots on the Visium slide. Spots with SRT data were histologically annotated from the HE-stained image, with 89 spots annotated as extra-adrenal connective tissue and excluded from further analysis (Fig. 4a). Additionally, 16 spots with a gene count of less than 1000 were excluded. Accordingly, downstream analysis was conducted using SRT data from 3380 spots (with a mean UMI of 38179 and a mean gene count of 7640).

Unsupervised clustering of the SRT spots resulted in 16 clusters, where spatially close spots were frequently assigned to the same clusters (Fig. S4a and b). The clusters were matched with histological annotations and merged into the Adj.AC, SPN, CPA, and capsule clusters (Fig. 4b, Fig. S4c–e, Table S9). The spatial distribution of the histological annotations and expression-based clusters was consistent, confirming that the classification of Adj.AC, SPN, and CPA is possible based on gene expression.

Differential expression analysis showed that the genes involved in adrenal androgen synthesis (*CYB5A* and *SULT2A1*) were upregulated in the SPN cluster (Fig. S4f, Table S10). MHC class II molecules (such as *HLA-DRA* and *HLA-DQA1*) were also upregulated in the SPN cluster. In the CPA cluster, the genes involved in cortisol synthesis (*CYP11A1*, *CYP21A2*, and *CYP17A1*) were upregulated. Enrichment analysis was performed for DEGs upregulated in each expression-based cluster (Fig. S4g, Table S11). The upregulated DEGs in the SPN cluster were enriched in gene sets related to immune response (“positive regulation of immune response” and “innate immune response”). These data were comparable to those obtained by the RNA-seq analysis (Fig. 3b).

### The two layers of SPNs have different gene expression profiles

To further characterise the two-layered structure of SPNs, we clustered the SPNs based on gene expression. Unsupervised clustering classified the SPN cluster into six subclusters matched with histological annotations and merged into either the zF-like SPN subcluster (SPN-F) or the zR-like SPN subcluster (SPN-R; Fig. 4c, Fig. S5a–d, Table S12). The spatial distributions of the histological annotations and expression-based SPN subclusters were generally consistent.

Differential expression analysis was performed for SPN-F and SPN-R. Regarding the gene expression of steroidogenic enzymes, *CYB5A* was significantly upregulated in SPN-R, consistent with the results of the IHC analysis (Fig. 4d, Fig. S5e, Table S13). In this study, expression levels of *HSD3B2* were not examined since the corresponding probe was a deprecated probe with off–target activity and was excluded during the Space Ranger processing of the sequencing data. Other genes such as *CCN3* (also known as *NOV*), *NCAM1*, and *PCP4*, were upregulated in SPN-F, while *FGG*, *TSPAN12*, and *PAH* were upregulated in SPN-R (Fig. 4e), consistent with the upregulation in zF and zR, as recently reported.^7^^,^^33^

We also performed an enrichment analysis of the upregulated genes and estimated pathway activities using the PROGENy method.^25^ In SPN-F, the upregulated genes were enriched in gene sets related to cell migration, such as “chemotaxis” and “positive regulation of epithelial to mesenchymal transition” (Fig. S5f, Table S14), with elevated p53 and Wnt pathway activities (Fig. 4f, Table S15). Meanwhile, in SPN-R, the upregulated genes were enriched in the gene sets related to immune response, such as “MHC class II protein complex assembly” and “inflammatory response” (Fig. S5f) with elevated NFκB and androgen pathway activities (Fig. 4f, Table S15). These observations suggest that the two-layered zF- and zR-like structures have distinct biological implications during adrenocortical tumorigenesis.

### Inference of developmental trajectory from SPNs to CPAs

**Fig. 5:**
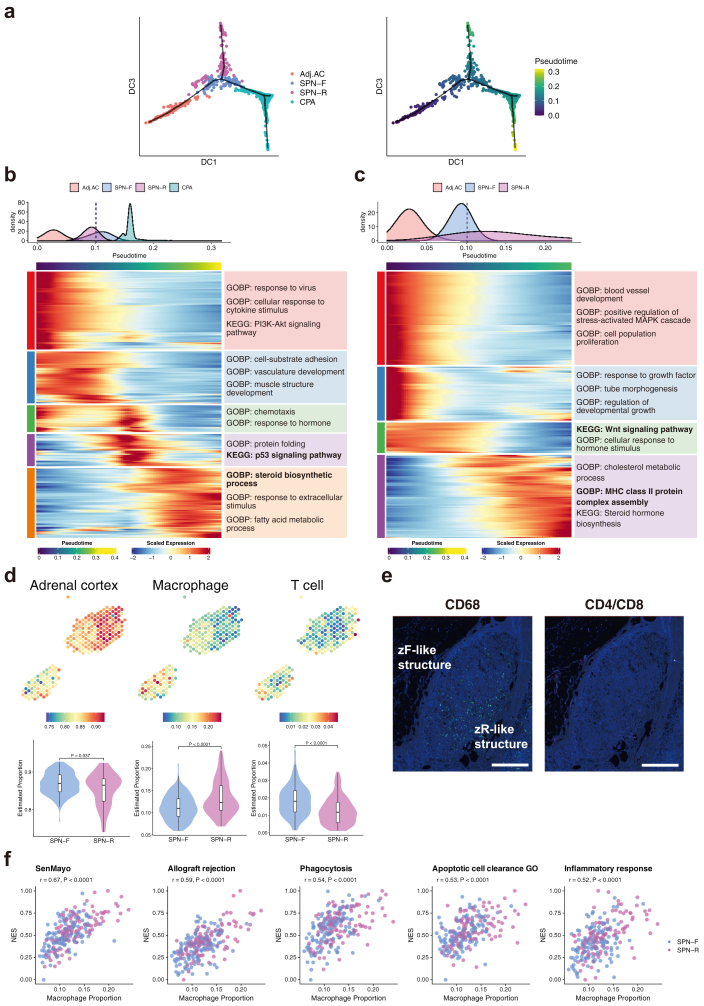
**Inference of developmental trajectory from SPNs to CPAs and characterisation of macrophages in SPNs. (a)** The result of the trajectory analysis projected onto a diffusion map. The dots are coloured by expression-based clusters in the left panel and by pseudotime in the right panel (**b** and **c**) Gene expression changes along pseudotime for CPA lineage (**b**) and SPN lineage (**c**). Top, density of expression-based clusters along pseudotime. The dashed line indicates the lineage branching point. Bottom, expression of DEGs along pseudotime axis for each lineage. Genes are clustered by k-means, and enriched GOBP/KEGG terms are shown on the right side of the heatmap. (**d**) The results of deconvolution analysis. The upper panel is a dot plot showing the spatial distribution of the estimated proportion of cells per spot, while the lower panel is a violin plot comparing SPN subclusters. (**e**) Immunofluorescence staining for CD68 (left) and CD4/CD8 (right) in SPNs. Scale bar, 500 μm. (**f**) The results of correlation analysis of macrophage proportion and enrichment scores of each gene set. Correlation coefficients were calculated by Spearman's test.

We performed trajectory analysis to infer the developmental trajectory of SPNs and CPAs based on gene expression. In this study, two lineages were inferred; one from Adj.AC leading to CPA via the SPN cluster (termed the CPA lineage) and one from the Adj.AC terminating at the SPN cluster (termed the SPN lineage; Fig. 5a). The two CPA and SPN lineages branched off at SPN-F and moved to CPA and SPN-R, respectively (Fig. S6a).

In the CPA lineage, the expression of steroidogenic genes (such as *CYP17A1*, *CYP11A1*, and *STAR*) increased along the pseudotime (Fig. 5b, Tables S16, S17). The expression of genes involved in protein folding (such as *HSPA1B*, *HSPA2*, and *DNAJB1*) and the p53 signalling pathway (such as *BAX*, *DDB2*, and *ZMAT3*) was upregulated during the transition from SPN-F to CPA, suggesting that SPN-F progresses to CPA with increased protein synthesis and cell proliferation. In contrast, the expression of genes related to cell proliferation (such as *BCL2*, *ITGA2*, and *WNT11*) and the Wnt signalling pathway (such as *WNT4*, *LEF1*, and *AXIN2*) was downregulated along the pseudotime in the SPN lineage (Fig. 5c). These observations, taken together, suggest that SPN-R arises from SPN-F due to reduced Wnt signalling.

### Macrophage-mediated immune response in the zR-like structure

Since genes related to immune response were upregulated in SPNs, particularly in SPN-R (Figs. 3b and 4f, Fig. S5f), we next examined the spatial distribution of immune cells in SPNs. Using previously reported single-cell RNA-seq data of normal adult adrenal tissues (Array Express E-MTAB-11837)^7^ as a reference, we performed deconvolution analysis to estimate the proportion of immune cells (macrophages and T cells) in each spot. The median proportion of cells per SPN spot was 11.5% for macrophages and 1.5% for T cells. The proportion of macrophages was higher in SPN-R than in SPN-F (median proportion SPN-F 11.0%, SPN-R 12.3%; Wilcoxon rank sum test, P value < 0.0001, median difference −1.7, 95% confidence interval [−2.6, −0.84]; Fig. 5d). These observations suggest that the immune cells in SPNs are mostly macrophages, with their distribution concentrated towards SPN-R. Using the PhenoCycler-Fusion System, we performed multiplex immunofluorescence imaging on an FFPE section from the same case (Case C-5).^28^ Macrophages, identified by CD68 positivity, were present in SPNs; their distribution was concentrated toward SPN-R, consistent with the data obtained through deconvolution analysis (Fig. 5e). In contrast, T cells, identified by CD4 and CD8 positivity, were rarely present in SPNs (Fig. 5e).

ssGSEA using the Hallmark gene sets in MSigDB showed that the proportion of macrophages was positively correlated with the enrichment scores of inflammation-related gene sets such as “allograft rejection,” “inflammatory response,” and “apoptosis” (Fig. 5f). Furthermore, ssGSEA using custom-curated gene sets by Wilmouth et al.^35^ showed that the proportion of macrophages is positively correlated with the enrichment scores of the phagocytosis- and senescence-related gene sets (Fig. 5f, Fig. S6b, Tables S18, S19). These observations, taken together, suggest that the zR-like structure of SPNs is involved in a macrophage-mediated immune response and senescence.

### High SPN-R signature in ACC is associated with better prognosis

**Fig. 6:**
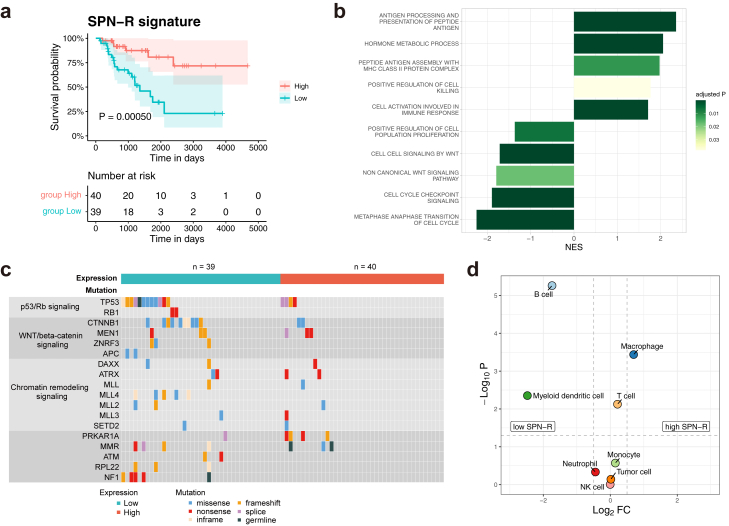
**Association of high SPN-R. signature with better prognosis in ACCs (a)** Survival analysis of 79 patients with ACC in the TCGA consortium divided into two groups, high (red) and low (blue), according to the expression of the SPN-R signature. Kaplan–Meier survival curves are shown with 95% confidence bands (shaded areas). Survival curves are compared by log-rank test, and the resulting P value is shown. (**b**) Gene set enrichment analysis comparing the gene expression of high and low SPN-R signature groups using gene ontology biological process gene sets. Selected gene sets are shown. The gene set with a positive normalised enrichment score indicates the enrichment of genes whose expression was upregulated in the high SPN-R signature group. (**c**) Different frequencies of mutations in ACCs between the high and low SPN-R signature groups. (**d**) Volcano plot showing the difference of inferred immune cell fraction between high and low SPN-R signature groups. The horizontal dashed line indicates the adjusted P value threshold of 0.05 (Wilcoxon rank sum test). The vertical dashed line indicates the log_2_ fold-change threshold of 0.5.

To explore the functional role of SPN-R during adrenocortical tumorigenesis, we examined whether the SPN-R signature (top 10 genes upregulated in SPN-R) is associated with the prognosis of patients with ACC using the data extracted from TCGA database. The 79 ACCs were divided into two groups based on the median expression levels of the top ten upregulated DEGs in SPN-R (Table S20). The high SPN-R signature group (positive Z-score) had a better prognosis than the low SPN-R signature group (negative Z-score; log-rank test, P value = 0.00050; Fig. 6a). The hazard ratio, estimated using the Cox proportional hazards model, was 0.22 (with a 95% confidence interval of 0.087–0.56; Fig. S7a). The proportional hazards assumption underlying Cox regression was valid from diagnostic plots (Fig. S7b).

Differential expression analysis and GSEA were performed on the high and low SPN-R signature groups. The upregulated genes in the high SPN-R signature group were enriched in the gene sets related to immune response, such as “peptide antigen assembly with MHC class II protein complex” and “positive regulation of cell killing” (Fig. 6b, Tables S21 and S22). Besides, the downregulated genes in the high SPN-R signature group were enriched in the “cell cycle checkpoint signalling” and “non-canonical Wnt signalling pathway” gene sets. In the genomic analysis of ACCs using the TCGA database, driver mutations such as those associated with the Wnt/β-catenin pathway and TP53 were less frequent in the high SPN-R signature group (20%) than in the low SPN-R signature group (56%; Fisher's exact test, P value = 0.0011, odds ratio 0.19, 95% confidence interval [0.071, 0.53]; Fig. 6c, Tables S21 and S22).

Deconvolution analysis was performed to evaluate differences in immune cell composition between the two groups. In the high SPN-R signature group, macrophages were the most dominant immune cells (6.3%), followed by T cells (5.7%). Meanwhile, in the low SPN-R signature group, T cells (4.9%) were the most dominant, and macrophages were the third most dominant (3.9%; Fig. 6d, Fig. S7c). The proportion of macrophages was significantly higher in the high SPN-R signature group than in the low SPN-R signature group (Fig. 6d). In this study, no significant difference in prognosis between the two groups was observed, as divided based on the curated “adrenal macrophage” gene set (Fig. S7d). The hazard ratio, estimated using the Cox proportional hazards model, was 0.67 (with a 95% confidence interval of 0.31–1.5; Fig. S7a). The proportional hazards assumption underlying Cox regression was valid from diagnostic plots (Fig. S7b).

## Discussion

CPAs are a leading cause of adrenocortical tumours, most of which are caused by somatic mutations in genes such as *GNAS* or *PRKACA*. However, how CPAs develop after adrenocortical cells acquire genetic mutations remains poorly understood. With no appropriate rodent models of spontaneous CPAs, it is essential to examine human adrenocortical samples. In this study, using multi-omics analysis, we succeeded in the identification of an adrenocortical nodular structure termed “SPNs”. Given that *GNAS* mutations found in SPNs are known as a driver mutation of CPAs,^2^^,^^3^ it is likely that SPNs are a precursor lesion of CPAs; CPA might arise from one of the SPNs in adrenocortical tissues.

There is considerable evidence that somatic clonal expansion in normal or noncancerous tissues occurs during aging and/or in response to environmental insults such as alcohol drinking and tobacco consumption, and chronic inflammation.^4^ It is, therefore, important to understand how adrenocortical cells with *GNAS* mutations clonally expand to form SPNs. The G protein subunit Gαs, which is encoded by *GNAS*, regulates various intracellular signalling pathways in response to G protein-coupled receptor activation. Evidence has suggested that gain-of-function mutations in *GNAS* are oncogenic/tumorigenic; they promote tumorigenesis in diverse organs such as gastrointestinal and pancreatic cystic tumours.^36^ In the adrenal cortex, pituitary-derived ACTH activates cAMP/PKA signalling via the G protein-coupled melanocortin-2 receptor pathway, thereby promoting adrenocortical cell proliferation and steroids synthesis, with both zF and zR cells especially being highly dependent on cAMP/PKA signalling activities.^37^^,^^38^ Therefore, the adrenocortical cells, which acquire gain-of-function mutations of *GNAS*, might obtain cell proliferative and autonomous steroidogenic capacities independently of ACTH stimulation to become the dominant clones in adrenocortical tissues, where they might form SPNs. It is noteworthy that all *GNAS* mutations in a case with CPA and two SPNs (Case C-5) are mutually independent, thus representing a typical example of parallel evolution.^39^^,^^40^ These observations, taken together, suggest that SPNs arise from an independent distinct origin as a result of positive selection of *GNAS*-mutated clones in adrenocortical tissues, rather than from a common clone that branched off from a single origin. In this study, SPNs are present in Adj.AC to non-CPA as well as in Adj.AC to CPA, suggesting that SPNs occur in the adrenocortical tissues, rather than as a result of CPA-induced hypercortisolaemia and/or absence of ACTH stimulation. Further studies are required to elucidate how a particular adrenocortical cell(s) with *GNAS* mutations expand to form SPNs during the early process of human adrenocortical tumorigenesis.

SPNs possess the gain-of-function mutations in *GNAS*, which are all known as one of the driver mutations of CPAs^2^^,^^3^ and steroidogenic capacities even in the absence of ACTH stimulation. This is similar to a notion that APMs are a precursor lesion of APAs; they both share somatic gene mutations that confer autonomous aldosterone-producing capacity.^6^ In this study, the developmental trajectory of SPNs and CPAs based on gene expression shows that the zF-like structure in SPNs is enriched in the TP53 and Wnt signalling pathways with increased expression of steroidogenic enzymes, suggesting that the zF-like structure of SPNs has a potential to expand and contribute to the formation of CPAs. It is, therefore, likely that SPNs represent a precursor lesion during the progression to CPAs with *GNAS* mutations. Interestingly, no somatic mutations in *PRKACA* were identified in SPNs. It is conceivable that cell populations that acquire *PRKACA* mutations progress rapidly to CPAs, without a histopathological defined nodular structure. The CPAs in Cases C-4 and C-6 harboured *PRKACA* mutations. In both cases, a cell population with *PRKACA* mutation and one with *GNAS* mutation might occur independently in the same adrenal cortex, and the cell population with *PRKACA* mutation progressed rapidly to CPA, while the one with *GNAS* mutation remains as SPN. Although no mutations were found in N3, it is possible that the lesion had disappeared in the sections from which DNA was extracted.

The principal role of adrenocortical zR cells is to generate adrenal androgens. On the other hand, it is worthwhile to note that androgen-producing adrenocortical adenomas are extremely rare.^41^ A previous study showed that adrenocortical cell proliferation is inhibited by the pharmacological administration of androgen and suggested that androgens affect adrenocortical remodelling *per se*.^42^ Furthermore, Lyraki et al. reported that upregulation of R-spondin-1, a secreted Wnt agonist, results in an excessive immune response and cortical thinning in male mouse adrenals and suggested that endogenous androgens regulate Wnt signalling, ultimately suppressing the ectopic proliferation of adrenocortical cells.^43^ In this study, the zR-like structure of SPNs showed the features of reduced Wnt signalling, increased expression of genes associated with androgen synthesis, senescence and inflammation, as well as macrophage accumulation. Given the better prognosis of the high SPN-R signature group compared with that of the low SPN-R signature group in patients with ACC, the zR-like structure might exert a negative effect on adrenocortical cell proliferation or even an anti-adrenocortical tumorigenic effect. These observations, taken together, suggest that androgens produced in the zR-like structure induce adrenocortical cell senescence in SPNs, which is followed by a macrophage-mediated immune response. This is supported by a recent report that androgen-dependent adrenocortical senescence is induced in *Znrf3* conditional knockout mice, a pre-tumour state model of ACC, in which macrophages are recruited to serve as tumour suppressors.^44^ It is, therefore, interesting to speculate that the zF-like structure is tumorigenic and antagonised by the zR-like structure during the progression from SPNs to CPAs. Further studies are required to elucidate how the zR-like structure is involved in the formation of CPAs.

Even in the absence of ACTH stimulation, SPNs occur as a two-layered zF- and zR-like structure in the atrophied Adj.AC to CPA, possibly due to activation of the cAMP/PKA pathway by *GNAS* mutations. This is supported by a recent report that in the mouse adrenal cortex, the constitutive cAMP/PKA pathway activation by adrenal-specific deletion of *PRKAR1A*, a gene encoding one of the regulatory subunits of PKA, induces the differentiation to the androgen-producing zR-like zone, which is normally absent in mice.^38^ In this study, the zF- and zR-like structures in SPNs showed steroidogenic enzyme profiles and gene expression patterns that are highly similar to those in the human adrenocortical zF and zR, respectively. It is, therefore, conceivable that SPNs recapitulate some aspects of the differentiation from zF to zR, and SPNs could be considered ectopic adrenocortical two-layered structures in atrophied adrenocortical tissues. The molecular mechanisms underlying human adrenocortical zonation, especially the differentiation from the outer zF into inner zR cells, have not yet been addressed; it should be partly because of marked species differences in adrenocortical function and structure between mice and humans. Indeed, most rodents possess no zR in adrenocortical tissues.^1^ Our data highlight the importance of the cAMP/PKA pathway in the differentiation from zF to zR in humans, thereby suggesting that SPNs offer a unique experimental model with which to assess the mechanisms underlying human adrenocortical cell differentiation *in vivo*.

According to the 2022 WHO Classification,^45^ adrenocortical proliferations are pathologically classified into three types: adrenocortical nodular disease, adrenocortical adenoma, and ACC, among which adrenocortical nodular disease is subcategorised into three subtypes; sporadic nodular adrenocortical disease, bilateral small nodular adrenocortical disease such as primary pigmented nodular adrenocortical disease and bilateral large nodular adrenocortical disease. The sporadic nodular adrenocortical disease is characterised by nonfunctional nodules, the remaining of which are often caused by pathogenic germline mutations. Based on the size of the nodules, steroidogenesis, and genetic background, SPNs should belong to the category of adrenocortical nodular disease, thus representing a unique adrenocortical nodular structure separate from these three subtypes.

There are a couple of limitations to this study. First, since not all the adrenal tissues removed at surgery were thin-sectioned, SPNs might be present in the adrenal cortices in which SPNs were not detected. Second, due to the small size of SPNs, we did not perform all kinds of analyses on each SPN, including genomic, pathological, and SRT analysis. Third, in SRT analysis, each individual measurement of one spot is contributed by multiple cells. The differential expression and trajectory analysis using the Visium data may be affected by the type and percentage of cells in one spot. In addition, the trajectory analysis performed in this study did not follow the progression of a single clone. Further studies with multiple cases are required to validate the results of this study. Fourth, the frequency of SPNs in cases with CPA and non-CPA should be interpreted with caution, since large odds ratio estimates and confidence limits suggest sparse-data bias.^46^ Finally, this study was conducted at a single centre, with potential self-selection bias. Due to the small sample size, future multi-centre studies with a large sample size are required.

In conclusion, this study suggests that adrenocortical cells, when *GNAS* is mutated, acquire proliferative and steroidogenic capacities to become dominant as a result of positive selection in adrenocortical tissues, where they expand clonally to form SPNs. SPNs exhibit a two-layered zF- and zR-like structure, where two distinct cell populations might differently contribute to adrenocortical tumorigenesis. Given that *GNAS* mutations found in SPNs are known as a driver mutation of CPAs,^2^^,^^3^ we postulate that SPNs are a precursor lesion of CPAs; CPA arise from one of the SPNs in adrenocortical tissues. Our data also help elucidate the molecular mechanisms underlying the layered structures of human adrenocortical tissues and adrenocortical tumorigenesis.

## Contributors

Conceptualisation: TF, HU. Methodology: TF, HU, NI, TO. Software: HU, NI, TO. Formal analysis: TF, HU, NI, TO. Investigation: TF, HU, NI, TO, MY-U, HK, MF, NU, HN, NK, YM, RS, TM and MS. Resources: MS, YS, ME, YOda, SO. Visualisation: TF, HU, NI, TO. Funding acquisition: HU, YOgawa. Project administration: HU, YOgawa. Supervision: HU, YOgawa. Writing—original draft: TF, HU, NI. Writing—review & editing: TF, HU, NI, YOgawa. HU, NI and TO have accessed and verified the underlying data. All authors reviewed and approved the final manuscript. HU had final responsibility for the decision to submit the manuscript.

## Data sharing statement

Codes are archived at Zenodo (https://doi.org/10.5281/zenodo.8058032). RNA-seq fastq files and count data are available on ArrayExpress (E-MTAB-13109), and Visium Space Ranger output files are available on ArrayExpress (E-MTAB-13127). Other datasets generated and analysed during this study are available from the corresponding author upon reasonable request.

## Declaration of interests

The authors have declared that no conflict of interest exists.

## Appendix A Supplementary data

Tables S1–S22

Graphical abstract Adrenocortical cells, when *GNAS* is mutated spontaneously, acquire proliferative and autonomous steroidogenic capacities to become dominant as a result of positive selection in adrenocortical tissues, where they expand clonally to form SPNs. SPNs exhibit a two-layered zF- and zR-like structure, where two distinct cell populations might contribute differently to adrenocortical tumorigenesis. Given that *GNAS* mutations found in SPNs are known as a driver mutation of CPAs, SPNs are a precursor lesion of CPA; CPA arise from one of the SPNs in adrenocortical tissues.
