# Supplementary material for: Steroids-producing nodules: a two-layered adrenocortical nodular structure as a precursor lesion of cortisol-producing adenoma
Source: eBioMedicine. 2024 Apr 2;103:105087. doi: 10.1016/j.ebiom.2024.105087 (PMC11121169; doi:10.1016/j.ebiom.2024.105087)
Supplement: Figs. S1–S7 [file mmc1.pdf]

**a**

**b**

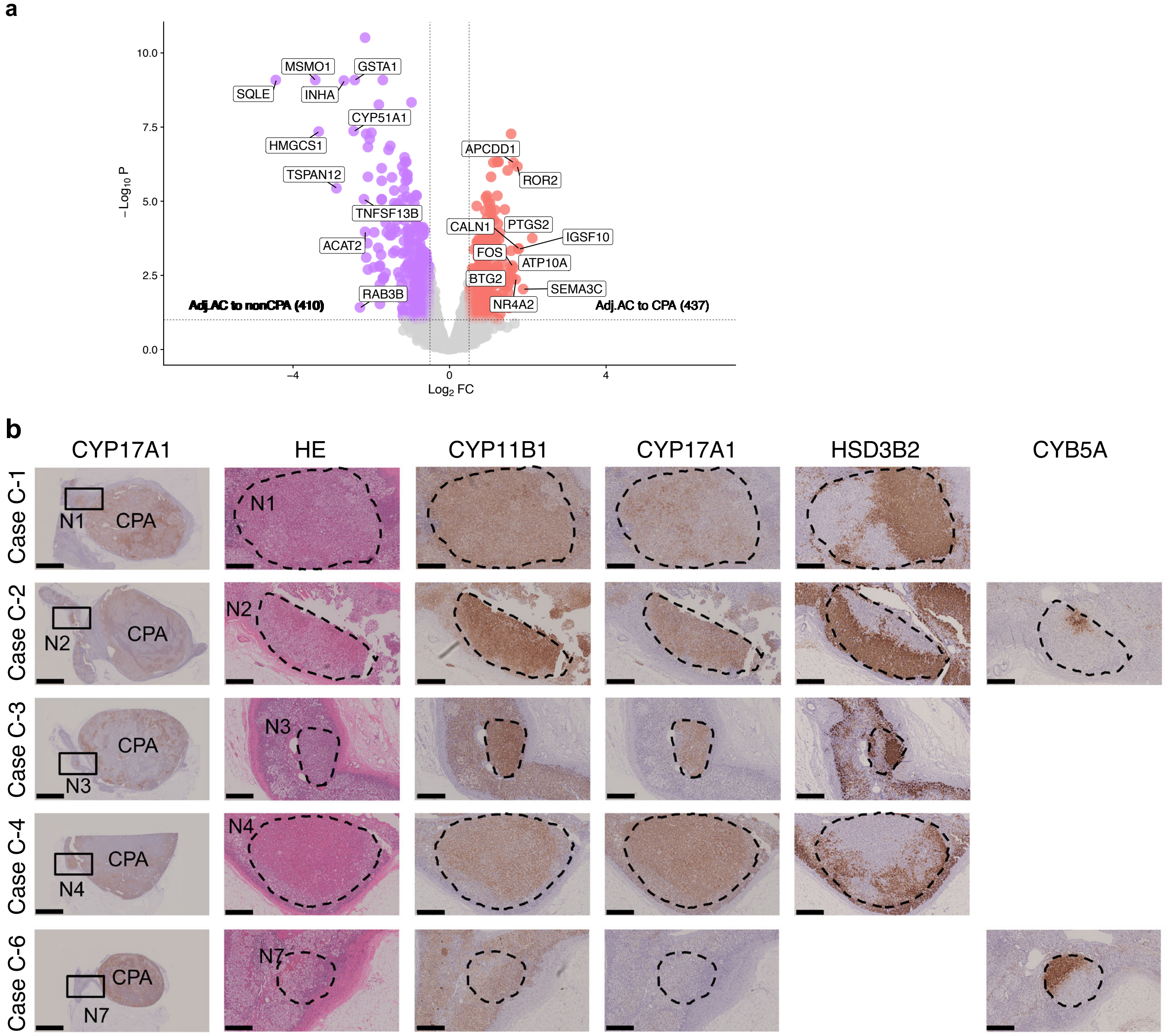


**Figure S1.** (**a**) Volcano plot showing differential gene expression between Adj.AC to CPA and non-CPA. The horizontal and vertical lines indicate the log2 fold-change threshold of 0.5 and the false discovery rate threshold of 0.1, respectively. Differentially expressed genes with the top 10 log2 fold changes in each group are highlighted. (**b**) Histological and immunohistochemical evaluation of SPNs (N1-N4, N7) in five cases. HE staining and immunohistochemistry of CYP11B1 and CYP17A1. In each case, the left image shows the entire adrenal gland and CPA (low magnification, 5 mm scale bar), and the other images show SPNs (high magnification, 1 mm scale bar).

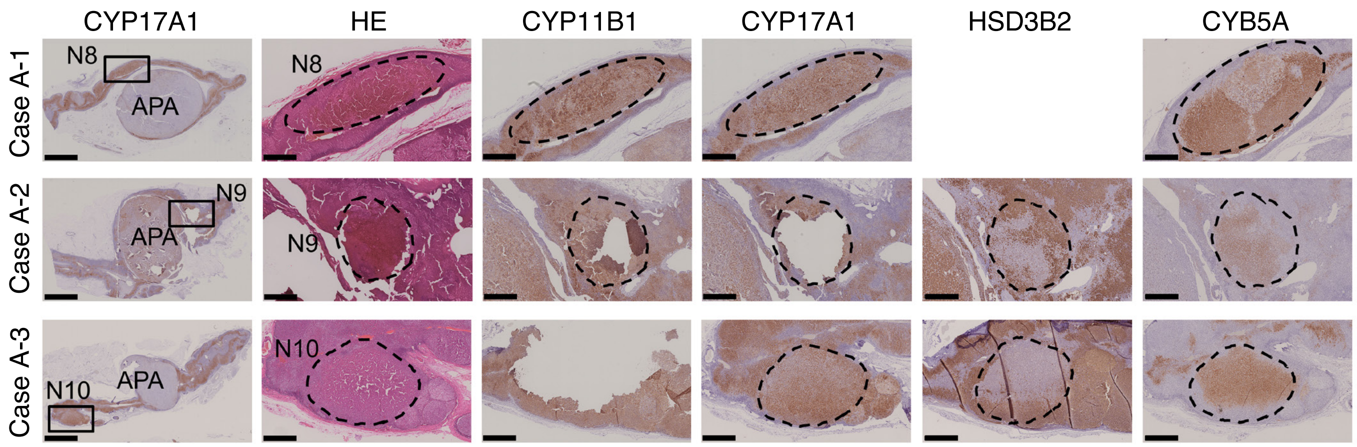


**Figure S2.** Histological and immunohistochemical evaluation of SPNs (N8-N10) in three cases of non-CPAs. HE staining and immunohistochemistry of CYP11B1 and CYP17A1. In each case, the left image shows the entire adrenal gland and adrenocortical tumour (low magnification, 5 mm scale bar), and the other images show SPN (high magnification, 1 mm scale bar).

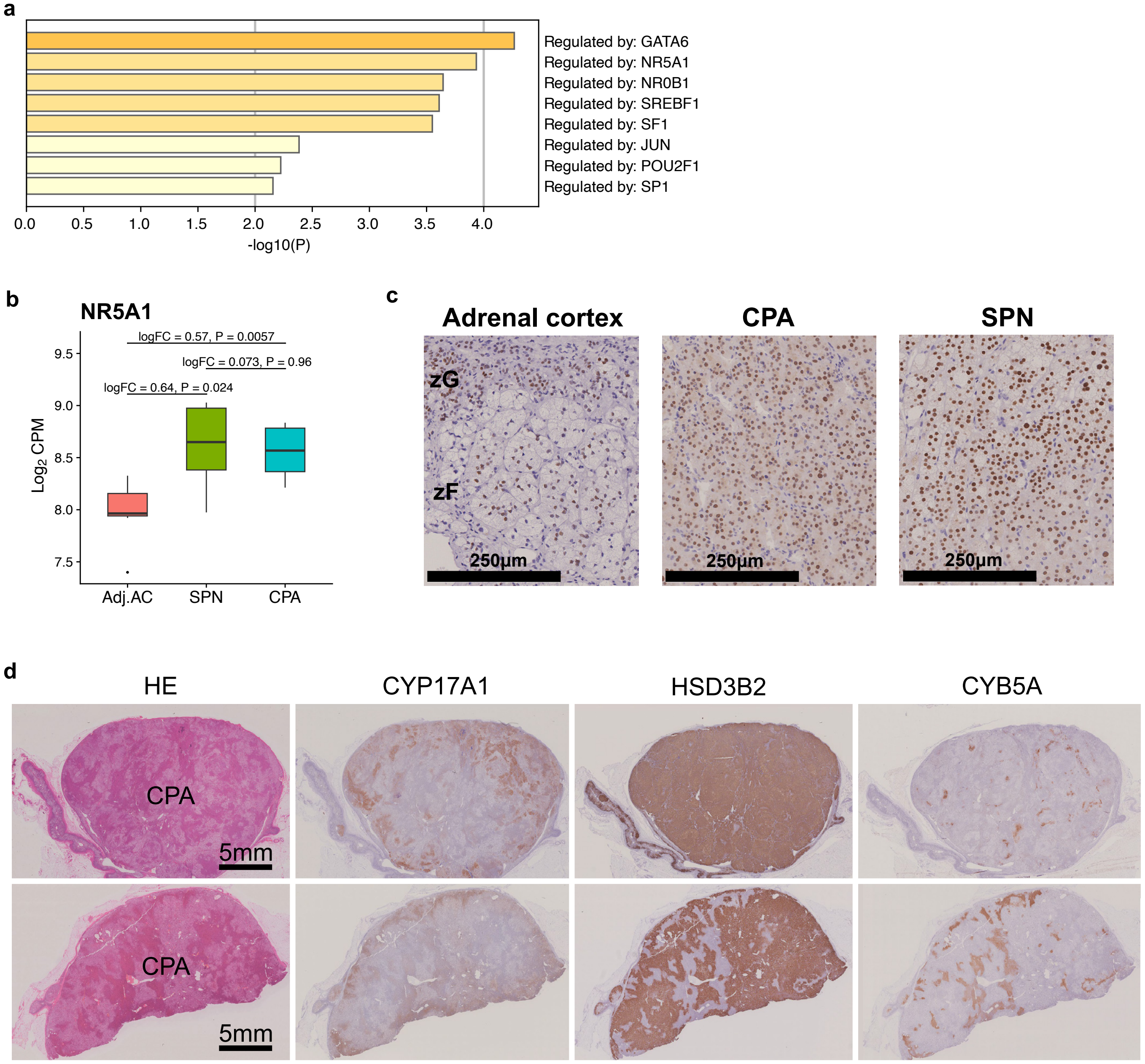


**Figure S3.** (**a**) Bar plot showing the result of TRRUST analysis. Transcription factors regulating genes commonly upregulated in CPA and SPN are inferred. (**b**) Boxplot comparing the expression of *NR5A1* in Adj.AC, CPAs, and SPNs. The outliers are defined as follows: Lower outliers are values less than Q1 − 1.5 × IQR, and upper outliers are values greater than Q3 + 1.5 × IQR (where IQR stands for Interquartile range, Q1 for the first quartile, and Q3 for the third quartile). (**c**) Representative histological images of immunochemistry analysis for SF1/Ad4BP in Adj.AC, CPA, and SPN. Scale bar, 250 μm. (**d**) Immunohistochemical evaluation of CPA with *PRKACA* somatic mutation (Upper images) and CPA with *GNAS* somatic mutation (lower images). HE staining and immunohistochemistry of CYP17A1, HSD3B2 and CYB5A. The zF-like component is stained with HSD3B2, and the zR-like component is stained with CYB5A. Scale bar, 5 mm. The upper images of HE and IHC staining are the same as the images of HE and IHC staining of Case C-3 in **Figure S1b**. The lower images of HE and IHC staining are the same as the images of HE and IHC staining of Case C-5 in **Figure 1d**.

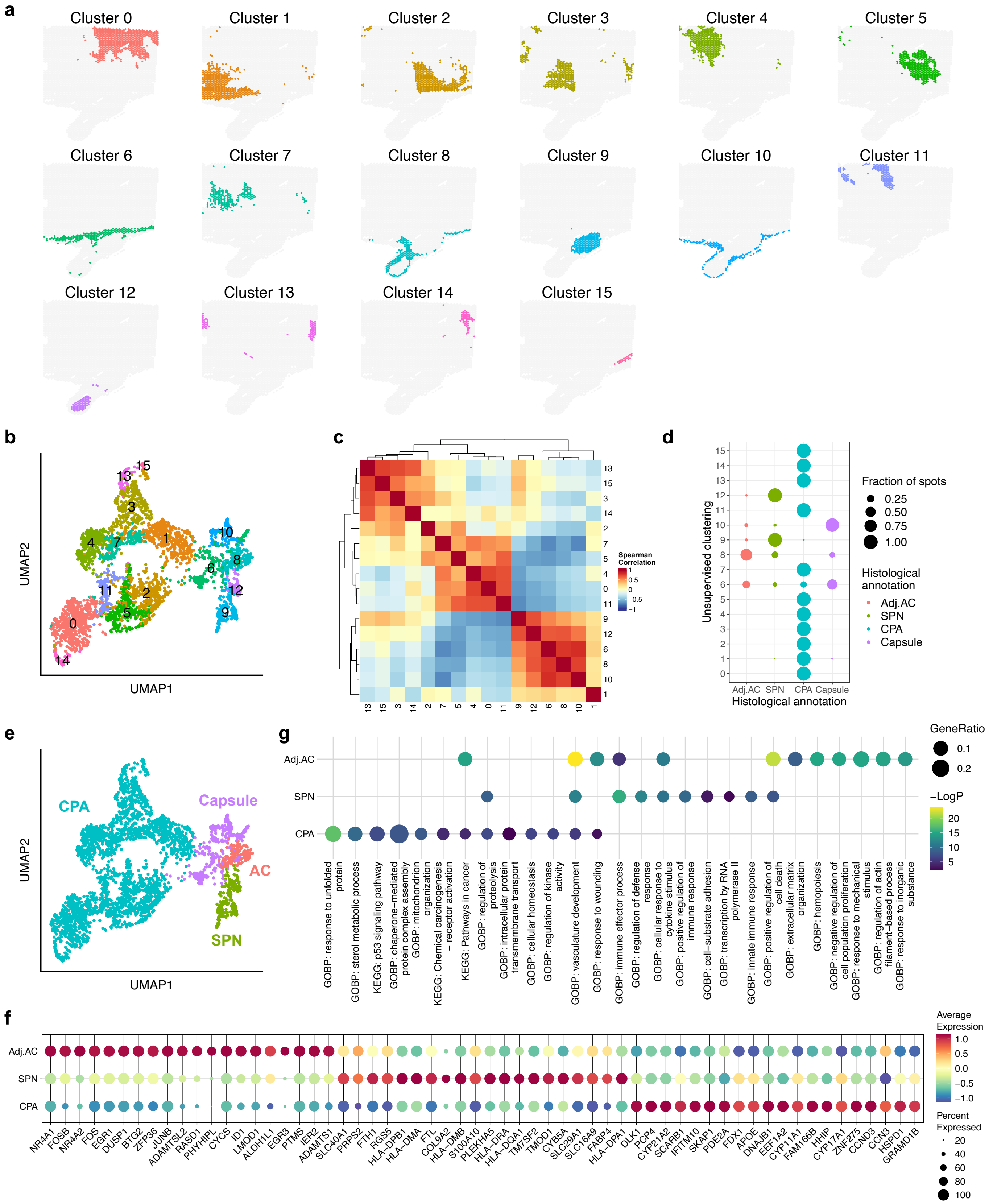


**Figure S4.** (**a**) Split view of the spatial distribution of each cluster classified by unsupervised clustering. (**b**) UMAP plots showing the results of unsupervised clustering based on gene expression. Dots represent SRT spots and colours represent clusters. (**c**) Correlation heatmap comparing the gene expression of clusters classified by unsupervised clustering. Clusters are ordered by hierarchical clustering of the Spearman correlation coefficients. (**d**) Dot plots showing the overlap between annotated regions and clusters classified by unsupervised clustering. The size of the dots represents the percentage of cluster spots belonging to the annotated regions. Histological annotations are shown on the x-axis and clusters on the y-axis. (**e**) UMAP plots showing clusters annotated with a combination of unsupervised clustering based on gene expression and histological annotation. Dots represent SRT spots and colours represent annotated cell types. (**f**) Dot plots showing the result of differential expression analysis. The top 20 log fold-change genes in each cluster are shown. (**g**) Dot plots showing the results of the GOBP and KEGG enrichment analysis. Terms enriched for upregulated genes in each cluster are summarized and ranked in order of decreasing P value. The top 10 terms are shown.

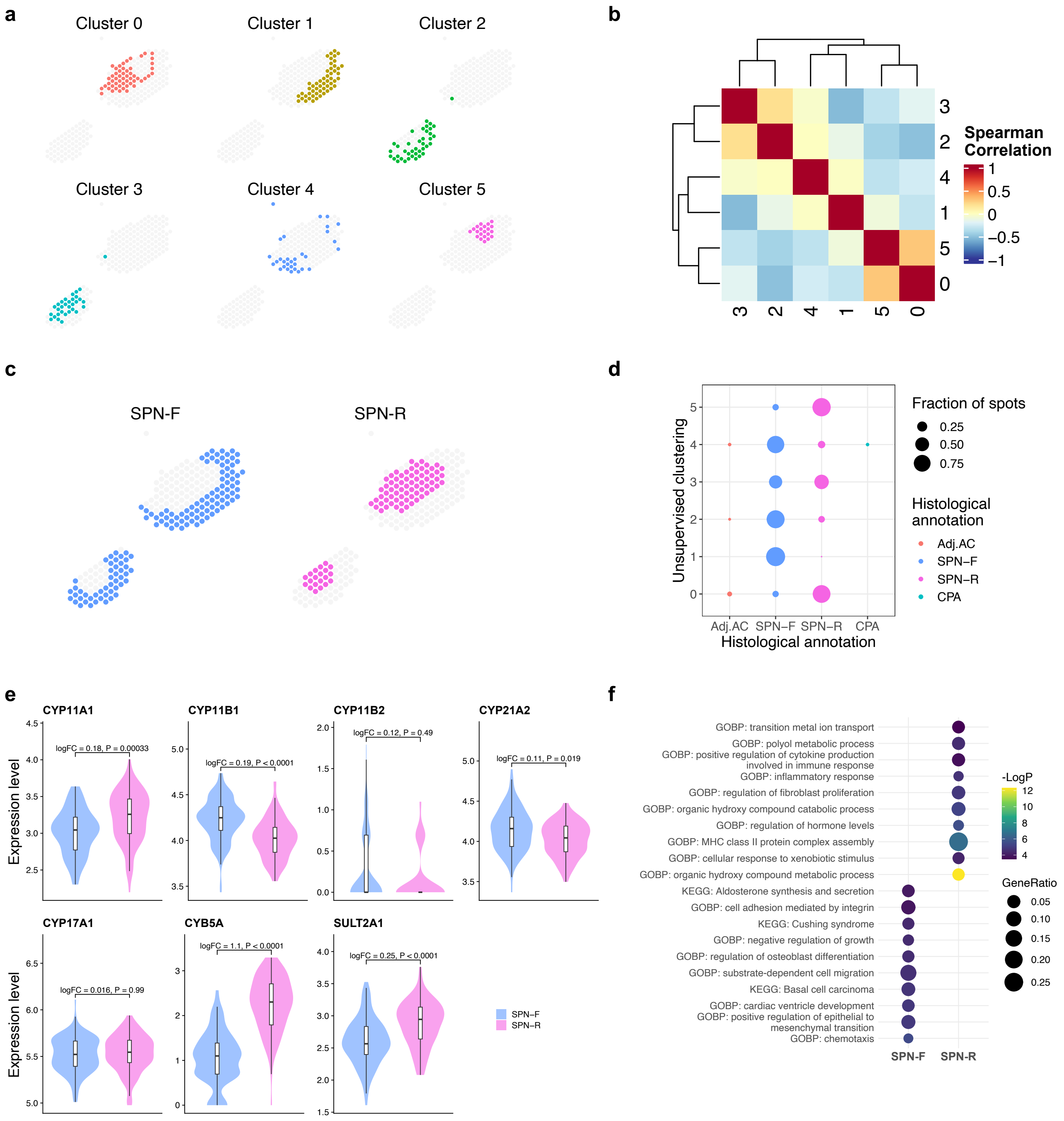


**Figure S5.** (**a**) Split view of the spatial distribution of each SPN subcluster classified by unsupervised clustering. (**b**) Correlation heatmap comparing the gene expression of clusters classified by unsupervised clustering. (**c**) Split view of the spatial distribution of each SPN subcluster classified by histological annotation. (**d**) Dot plots showing the overlap between annotated regions and SPN subclusters classified by unsupervised clustering. The size of the dots represents the percentage of cluster spots belonging to annotated regions. Histological annotations are shown on the x-axis and clusters on the y-axis. (**e**) Violin plots showing gene expression of steroidogenic enzymes. (**f**) Dot plots showing the results of the GOBP and KEGG enrichment analysis. Terms enriched for upregulated genes in each SPN subcluster are summarized and ranked in order of decreasing P value. The top 10 terms are shown. GeneRatio is the number of genes of interest in the gene set divided by the total number of genes in the gene set.

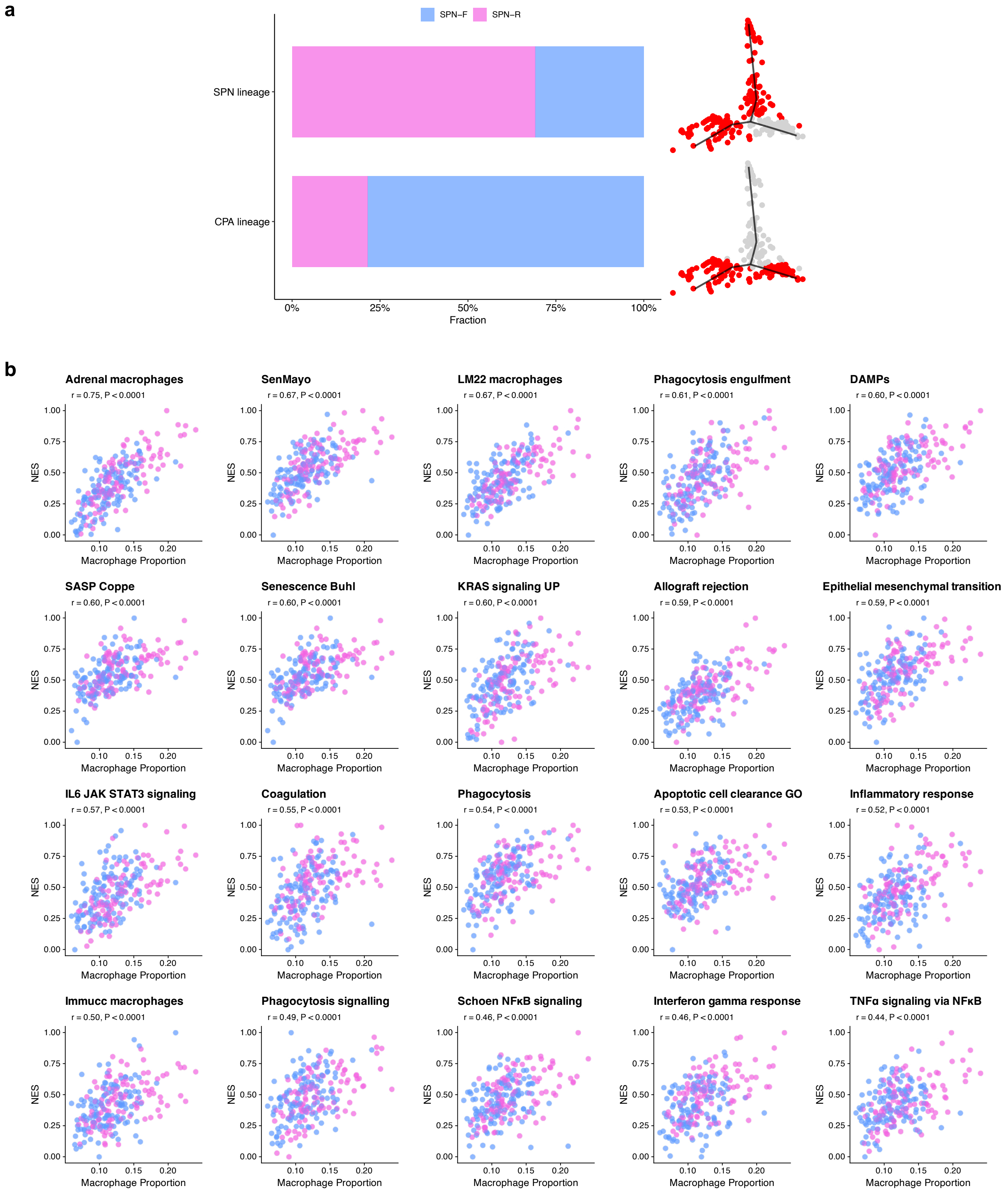


**Figure S6.** (**a**) Bar plots showing the percentage of SPN subclusters in the CPA and SPN lineages. The right side of the bar plots is a dot plot with only the SPNs from **Figure 5a** extracted. The red dots represent the spots of each lineage. (**b**) The results of correlation analysis of macrophage proportion and enrichment scores of each Hallmark gene set. Correlation coefficients were calculated by Spearman's test.

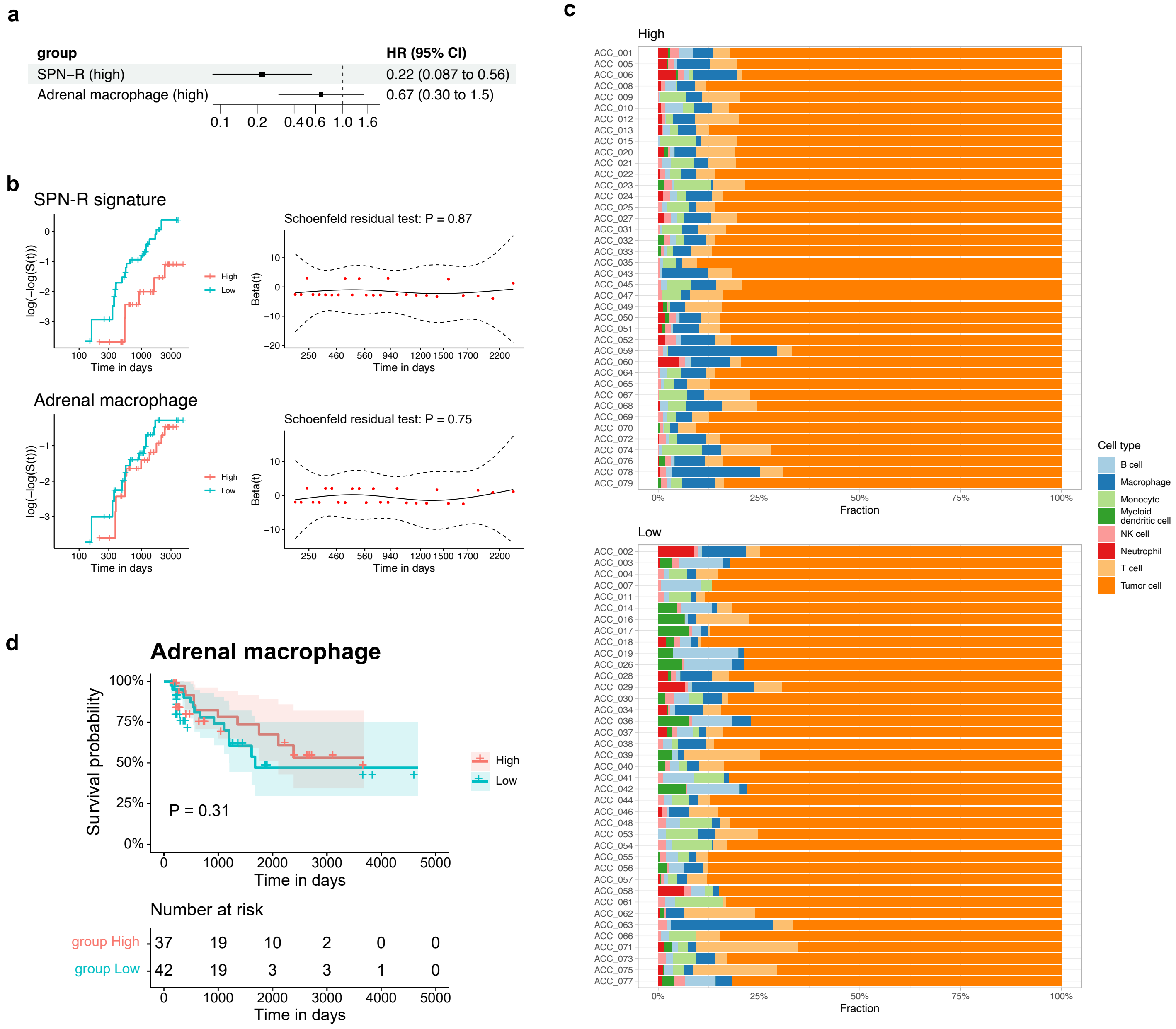


**Figure S7.** (**a**) Forest plot of hazard ratios for groups with higher expression of SPN-R signature and adrenal macrophage gene set. Hazard ratios and 95% confidence intervals were estimated using the Cox proportional hazards model. The x-axis is log-transformed. (**b**) Diagnostic plots to assess the proportional hazards assumption underlying the Cox regression for survival analyses. Upper left panel: log-log survival curves for the high (red) and low (blue) expression of SPN-R signature groups. Upper right panel: scaled Schoenfeld residuals of the Cox proportional hazards model for the SPN-R signature groups. Bottom left panel: log-log survival curves for the high (red) and low (blue) expression groups for the Adrenal macrophage gene set. Bottom right panel: scaled Schoenfeld residuals of the Cox proportional hazards model for the Adrenal macrophage gene set groups. (**c**) Bar plots showing the results of deconvolution analysis with TIMER 2.0. The colour of the bars indicates the estimated ratio of immune cells to tumour cells. (**d**) Survival analysis of 79 patients with ACC in the TCGA consortium divided into two groups, high (red) and low (blue), according to the expression level of the adrenal macrophage gene set. Kaplan-Meier survival curves are shown with 95% confidence bands (shaded areas). Survival curves are compared by log-rank test, and the resulting P value is shown.
