# Supplementary figures and images for: Steroids-producing nodules: a two-layered adrenocortical nodular structure as a precursor lesion of cortisol-producing adenoma
Source: eBioMedicine. 2024 Apr 2;103:105087. doi: 10.1016/j.ebiom.2024.105087 (PMC11121169; doi:10.1016/j.ebiom.2024.105087)

Graphical abstract

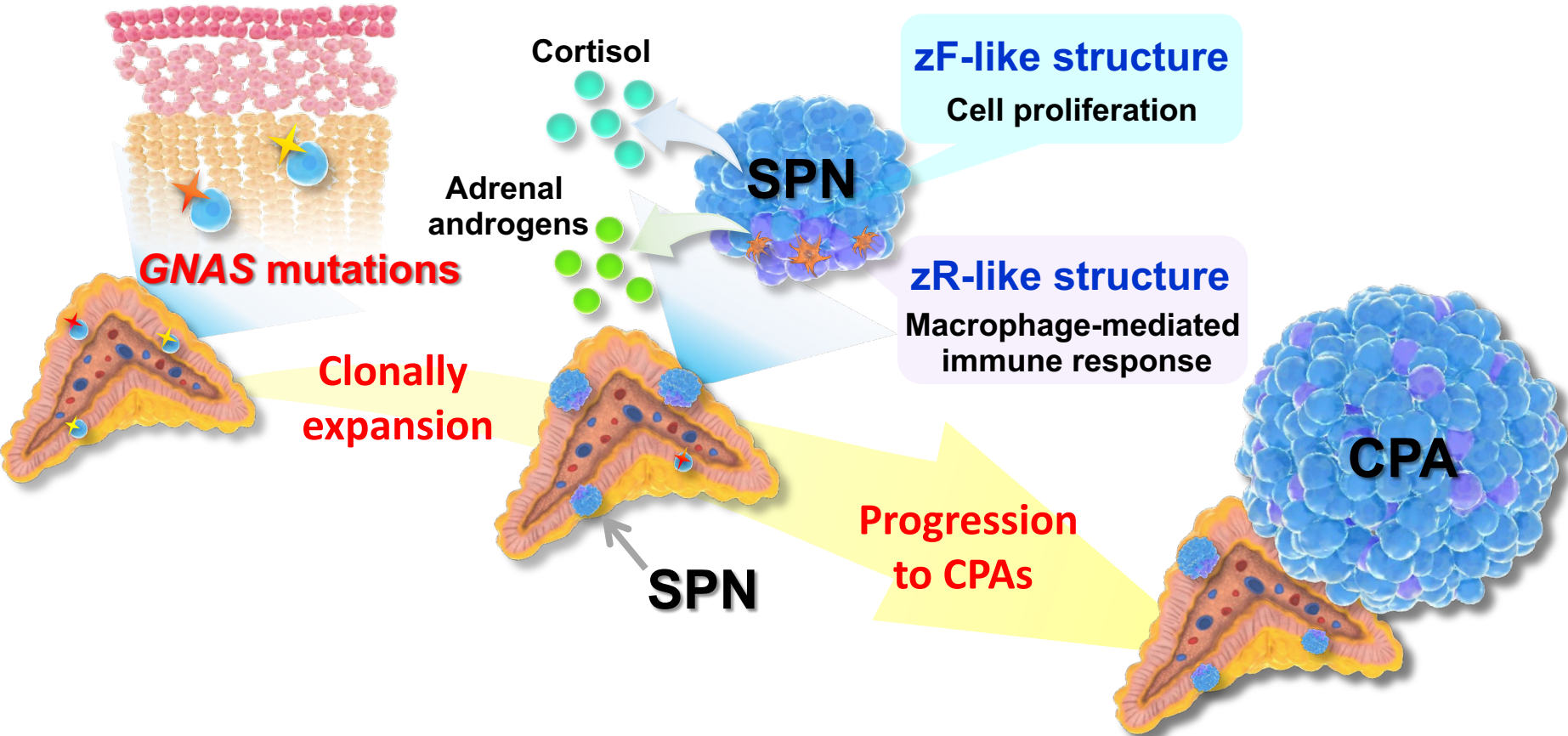

**Figure 1**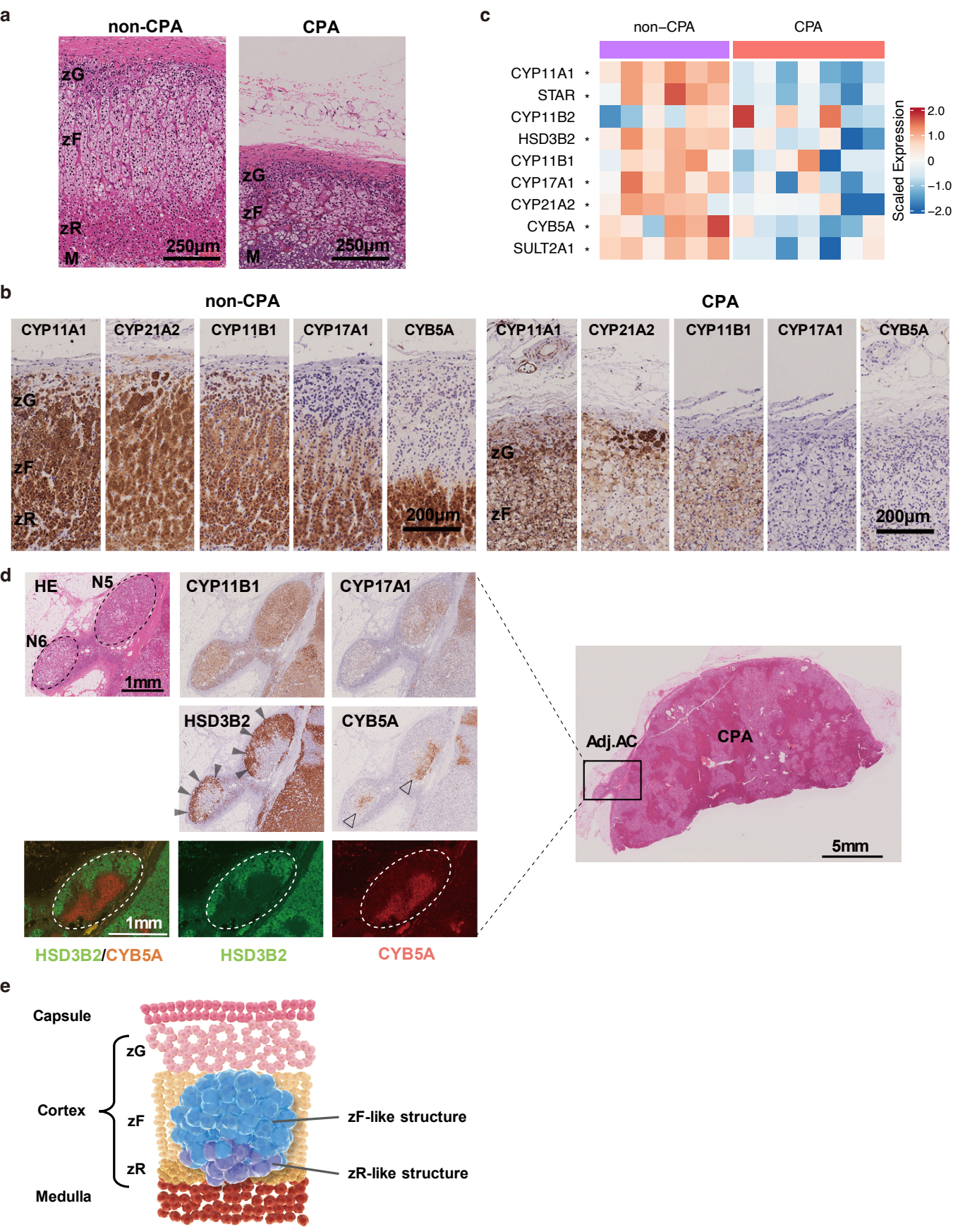

**Figure 2**

**a**

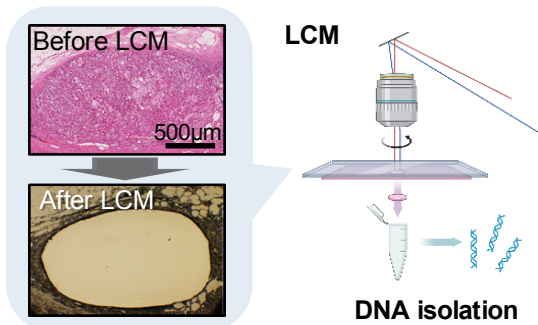

**b**

**GNAS (NM\_000516)**

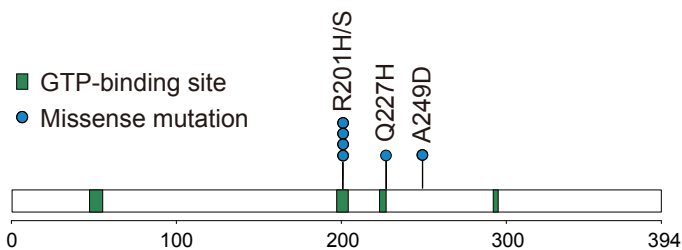

**c**

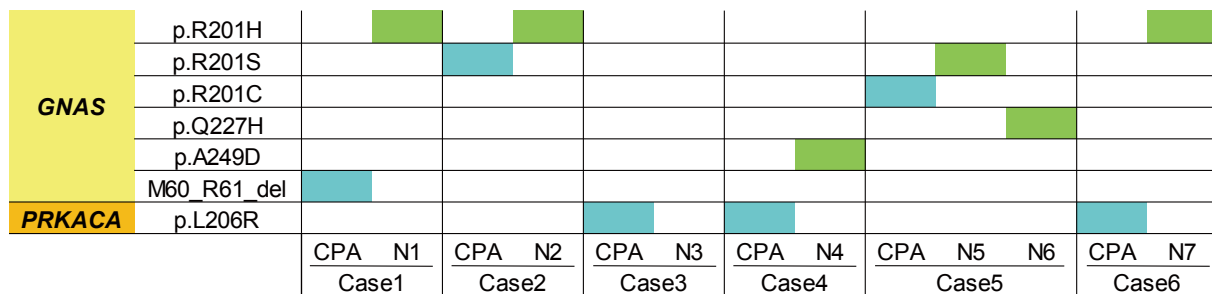

**d**

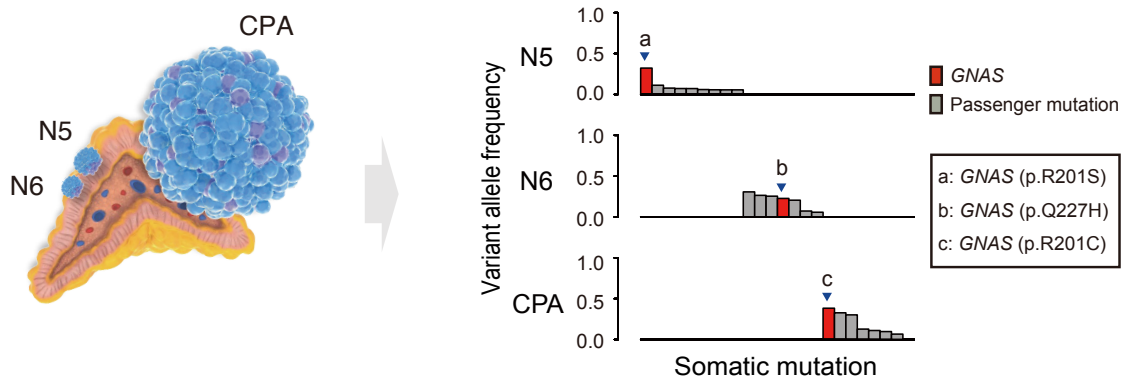

**e**

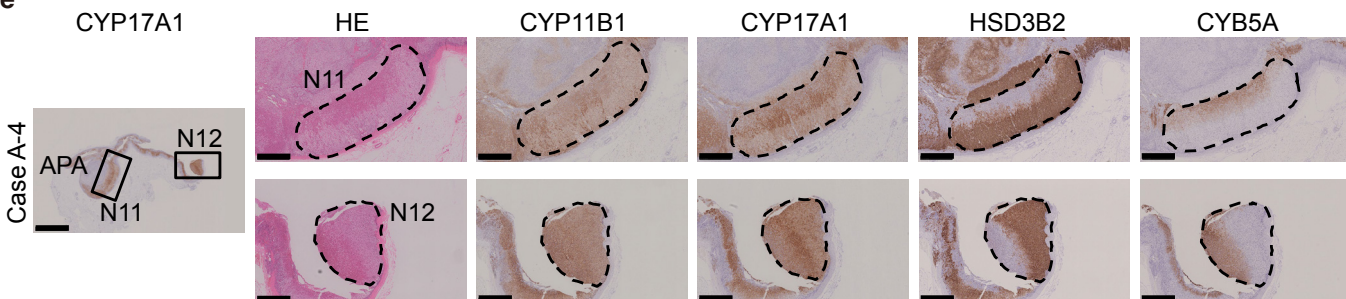

**Figure 3**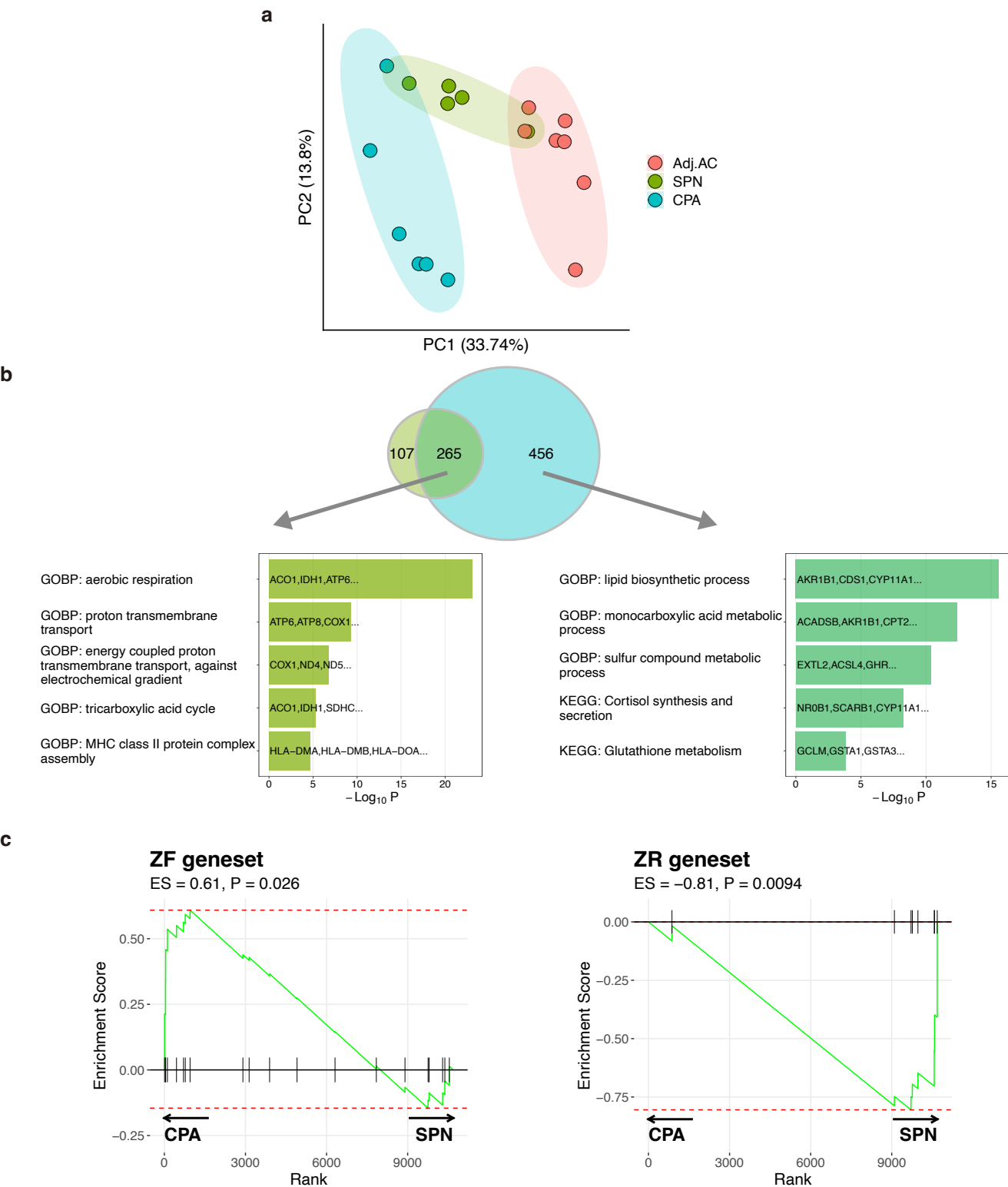

**Figure 4**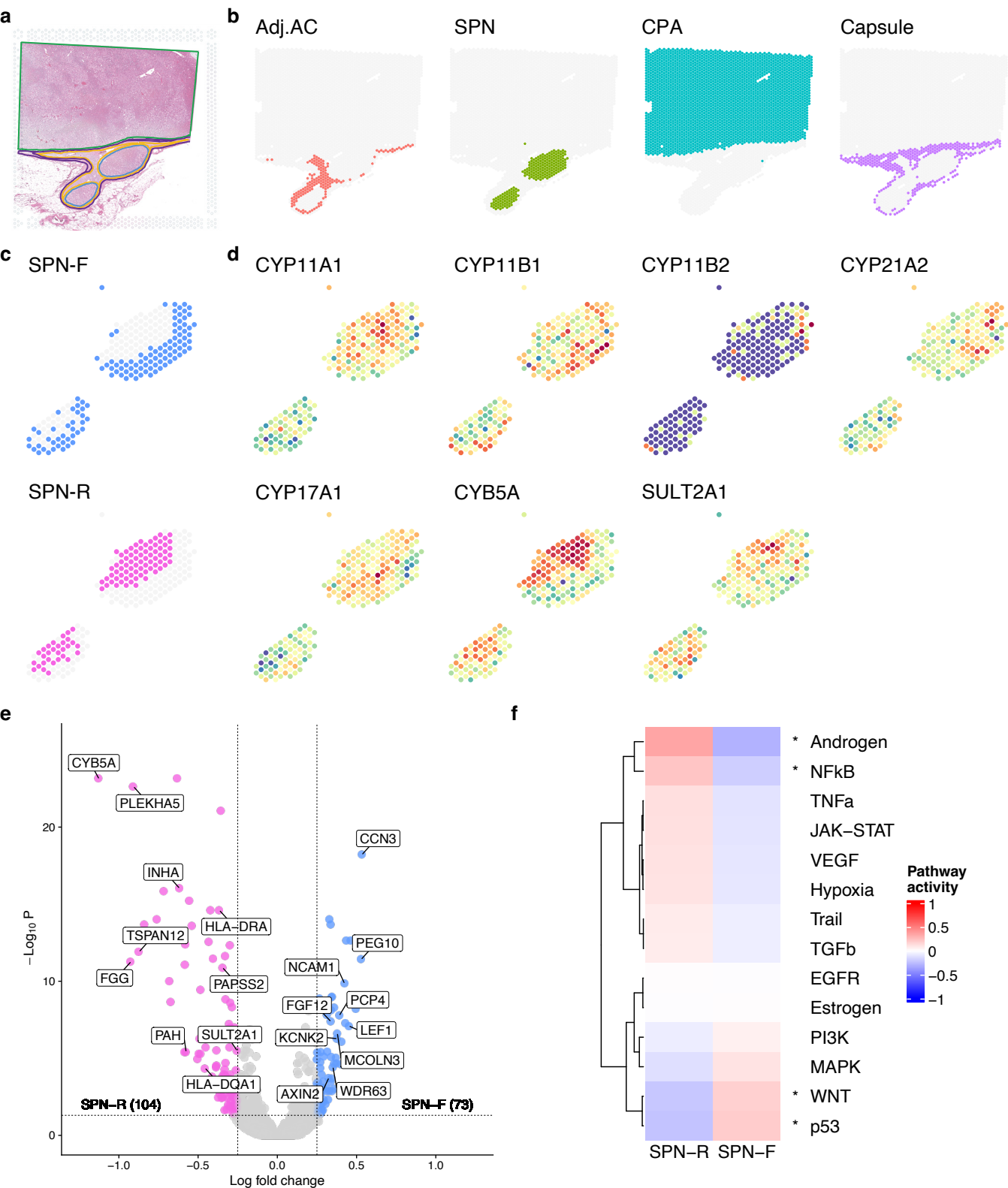

**Figure 5****a**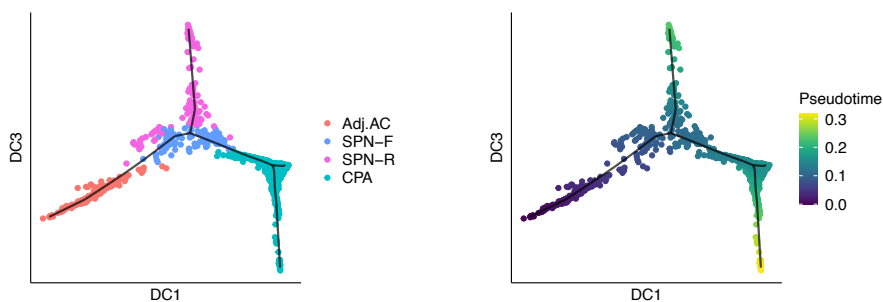**b**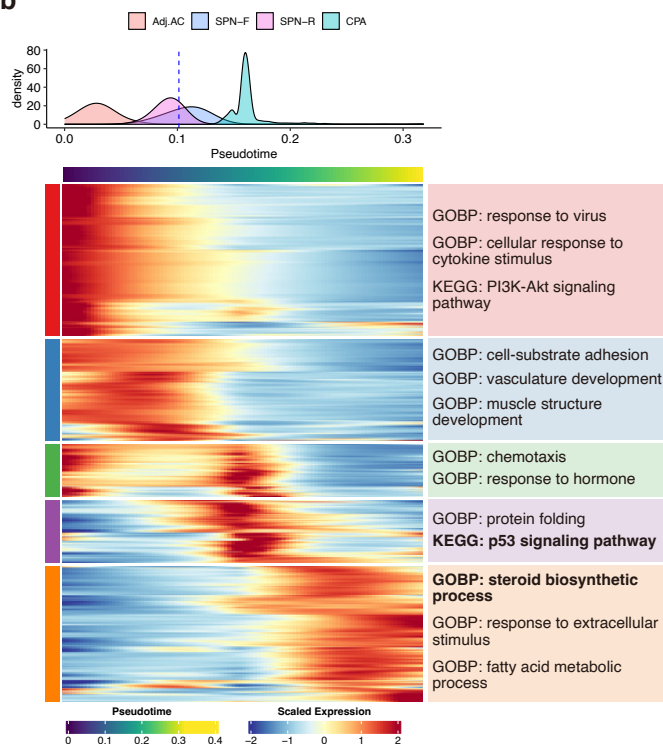**c**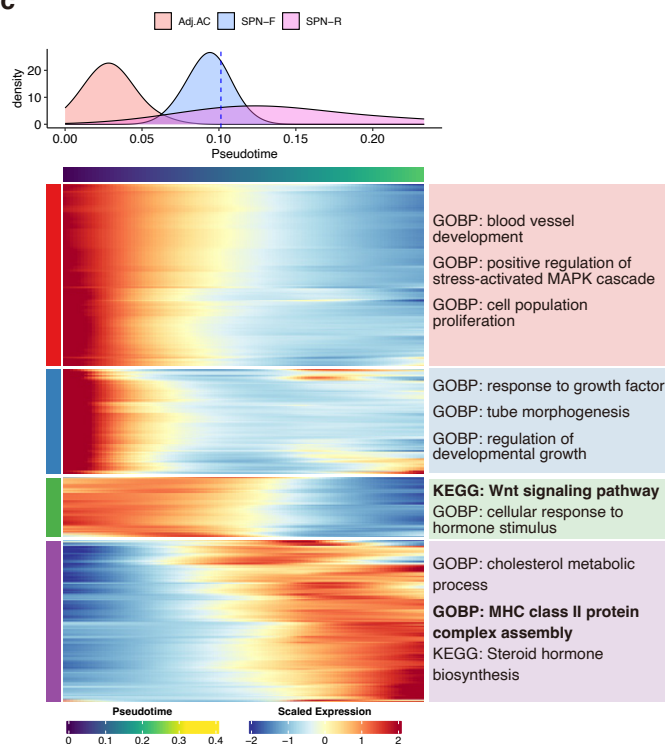**d**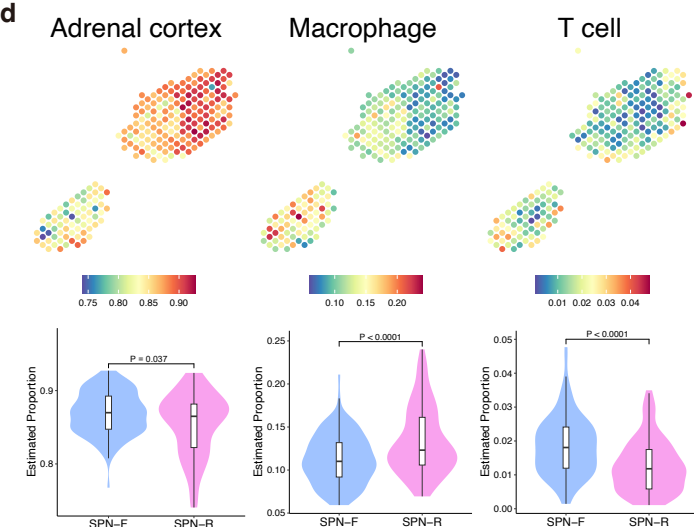**e**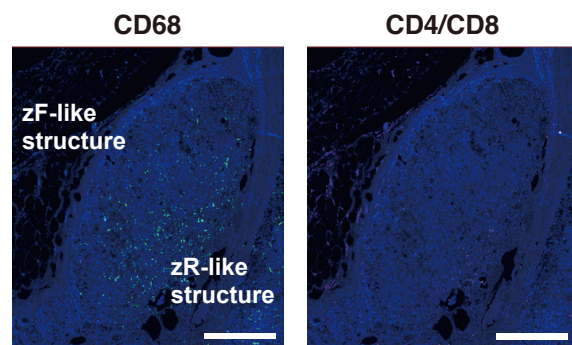**f**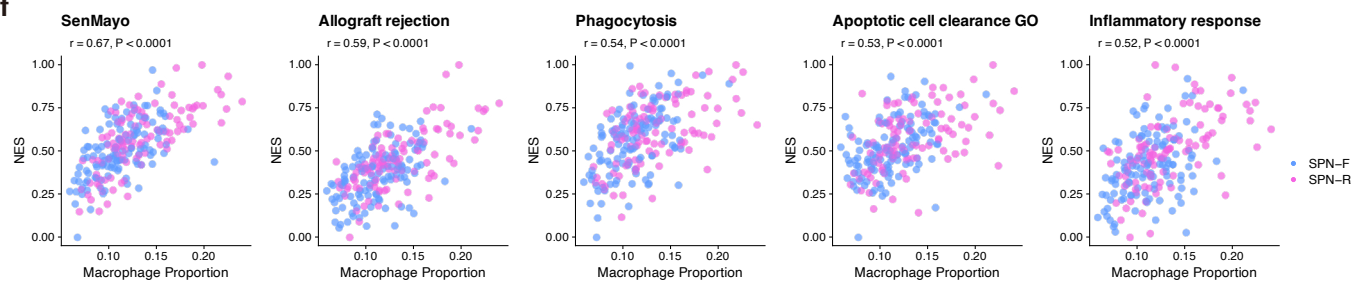

**Figure 6****a**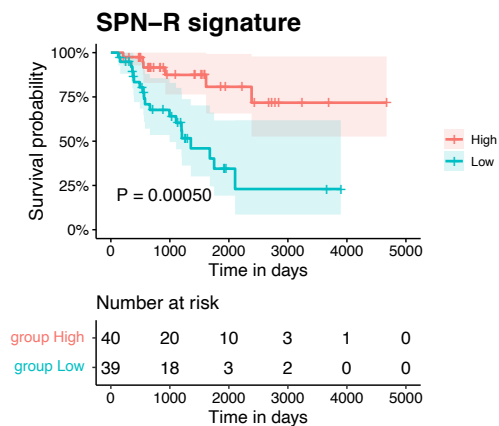**b**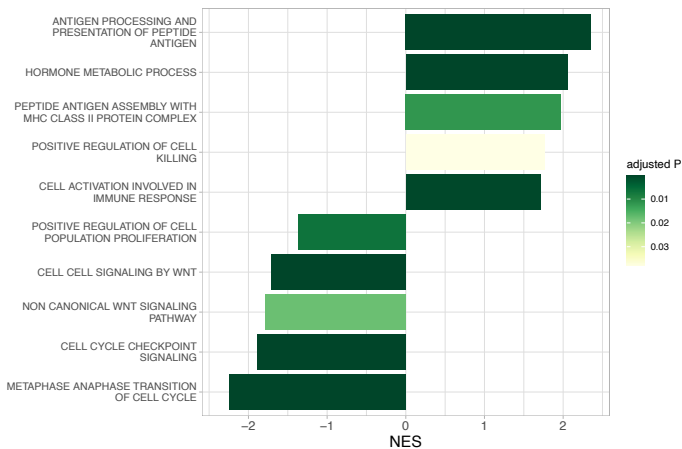**c**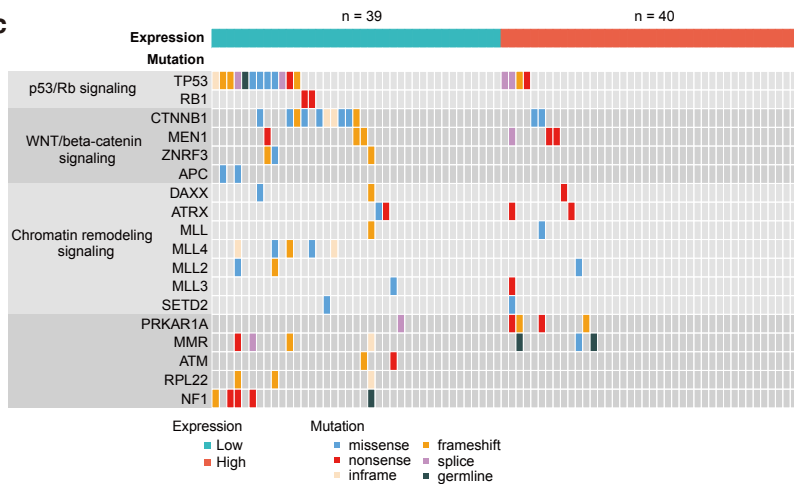**d**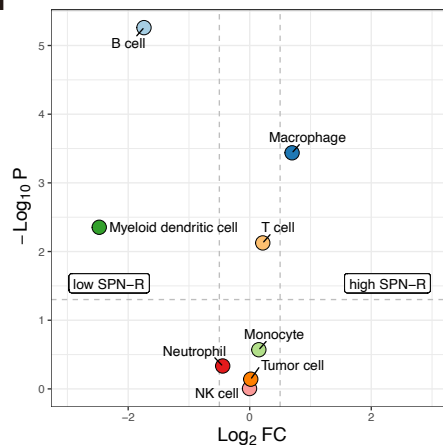

Supplement: Graphical abstract — Adrenocortical cells, when GNAS is mutated spontaneously, acquire proliferative and autonomous steroidogenic capacities to become dominant as a result of positive selection in adrenocortical tissues, where they expand clonally to form SPNs. SPNs exhibit a two-layered zF- and zR-like structure, where two distinct cell populations might contribute differently to adrenocortical tumorigenesis. Given that GNAS mutations found in SPNs are known as a driver mutation of CPAs, SPNs are a precursor lesion of CPA; CPA arise from one of the SPNs in adrenocortical tissues. [file mmc3.pdf]
